# A novel long non-coding RNA MIR4500HG003 promotes tumor metastasis through miR-483-3p-MMP9 axis in triple-negative breast cancer

**DOI:** 10.1038/s41419-024-06675-w

**Published:** 2024-05-02

**Authors:** Wen-Der Lin, Chia-Hao Chang, Jhih-Kai Pan, Forn-Chia Lin, Yu-Chia Chen, Ya-Jyun Chen, Po-Shun Wang, Wei-Qiao Hong, Sheng-Yuan Chen, Cheng-Han Lin, Yao-Lung Kuo, Wei-Pang Chung, Hui-Chuan Cheng, Michael Hsiao, Chia-Ning Yang, Pei-Jung Lu

**Affiliations:** 1 Institute of Clinical Medicine, College of Medicine, National Cheng Kung University, Tainan, Taiwan, ROC; 2 Department of Radiation Oncology, National Cheng Kung University Hospital, Tainan, 70401 Taiwan, ROC; 3 Division of General Surgery, Department of Surgery, Kaohsiung Veterans General Hospital, Kaohsiung, Taiwan, ROC; 4 Institute of Precision Medicine, National Sun Yat-sen University, Kaohsiung, Taiwan, ROC; 5 Department of General Surgery, National Cheng Kung University Hospital, Tainan, Taiwan, ROC; 6 Department of Oncology, National Cheng Kung University Hospital, Tainan, Taiwan, ROC; 7 Genomics Research Center, Academia Sinica, Taipei, Taiwan, ROC; 8 Department of Biochemistry, College of Medicine, Kaohsiung Medical University, Kaohsiung, Taiwan, ROC; 9 Department of Clinical Medicine Research, National Cheng Kung University Hospital, Tainan, Taiwan, ROC

**Keywords:** Breast cancer, Cell invasion

## Abstract

Breast cancer (BC) is the most common cancer and the leading cause of cancer-related deaths in women worldwide. The 5-year survival rate is over 90% in BC patients, but once BC cells metastasis into distal organs, it is dramatically decreasing to less than 30%. Especially, triple-negative breast cancer (TNBC) patients usually lead to poor prognosis and survival because of metastasis. Understanding the underline mechanisms of TNBC metastasis is a critical issue. Non-coding RNAs, including of lncRNAs and microRNAs, are non-protein-coding transcripts and have been reported as important regulators in TNBC metastasis. However, the underline mechanisms for non-coding RNAs regulating TNBC metastasis remain largely unclear. Here, we found that lncRNA MIR4500HG003 was highly expressed in highly metastatic MDA-MB-231 TNBC cells and overexpression of MIR4500HG003 enhanced metastasis ability in vitro and in vivo and promoted MMP9 expression. Furthermore, we found MIR4500HG003 physically interacted with miR-483-3p and reporter assay showed miR-483-3p attenuated MMP9 expression. Importantly, endogenous high expressions of MIR4500HG003 were correlated with tumor recurrence in TNBC patients with tumor metastasis. Taken together, our findings suggested that MIR4500HG003 promotes metastasis of TNBC through miR-483-3p-MMP9 signaling axis and may be used as potential prognostic marker for TNBC patients.

## Introduction

Breast cancer (BC) is the top-ranked diagnosed cancer in the world [1] and the second leading cause of cancer-related deaths among females in the United States [2]. BC can be classified into various subtypes, including luminal type (ER+/PR+/HER2−/+), HER2-enriched (ER-/PR-/HER2+), and triple-negative breast cancer (ER-/PR-/HER2-, TNBC) [3]. Targeted therapies including hormone therapy and immunotherapy have the potential to improve the survival rates of luminal and HER2-enriched BC patients [4, 5]. However, TNBC, which comprises 15–20% of BC cases, lacks specific targeted therapies due to the absence of these receptors [3, 6]. Moreover, TNBC is characterized by its highly aggressive form. Distant metastasis is observed in ~46% of TNBC patients [6], contributing to a poorer prognosis and reducing the 5-year survival rate to 12% [7, 8]. However, the underlying mechanism behind the high risk of distant metastasis in TNBC patients remains unclear.

The transition states of Epithelial-Mesenchymal Transition (EMT) have been shown to play a significant role in tumor initiation, invasion, metastasis, and even resistance to therapy. EMT-Transcriptional factors that participate in organizing EMT programs include ZEB1, ZEB2, SNAIL1, SNAIL2, and TWIST [9, 10]. During the process of EMT, numerous proteins participate in a complex collaboration. Activation of EMT leads to the loss of E-cadherin and certain cytokeratins, which are crucial components of the cytoskeleton, while N-cadherin, Vimentin, fibronectin, and β1 and β3 integrins are upregulated as replacements. The Matrix metalloproteinase (MMP) family of enzymes also play a significant role in facilitating cell migration and invasion through the degradation of the basement membrane.

Long non-coding RNA (lncRNA) is recognized definition as composition of more than 200 nucleotides [11, 12]. It has been indicated that lncRNAs participate in various physiological activities, including growth, metabolism, and homeostasis. Certain lncRNAs have been implicated in influencing tumor proliferation, survival, metastasis, and drug resistance. Given their critical roles in cancer development, lncRNAs hold great potential as therapeutic targets for cancer treatment [11, 12]. According to current studies, the cellular mechanisms of lncRNA can divide into five main pathways: (1) lncRNAs encode polypeptides; (2) lncRNAs participant in transcriptional regulation; (3) lncRNAs are involved in post-transcriptional regulation; (4) lncRNAs are implicated in epigenetic regulation; (5) lncRNAs act as a signal transducer [13]. For example, in post-transcriptional regulation, LINC008999 functions as a tumor suppressor by competitively sponging to miR-425, which can bind on 3’ UTR of DICR1 mRNA to induce degradation [14]. In terms of epigenetic regulation, lncRNA BLAT1 enhances promoter CpG methylation of miR-125b, which in turn inhibits ERBB2 degradation and contributes to BC development [15].

Here, we hypothesized that lncRNAs may target specific signaling pathway to mediate distant metastasis that leads to poor prognosis of TNBC patients. From RNA sequencing (RNA-Seq) data, we found that MIR4500HG003 was highly expressed in invasive MDA-MB-231 1-5 cells and overexpression of MIR4500HG003 enhanced cell migration and invasion ability. Furthermore, human metastatic mouse models of MIR4500HG003-overexpressed cells showed an increase of distant metastasis. Moreover, we demonstrated that MIR4500HG003 physically interacted with miR-483-3p in MS2-TRAP assay, as well as high level of MIR4500HG003 increased MMP9 level and gelatinase activity. Importantly, endogenous high expression of MIR4500HG003 was correlated with tumor recurrence in patients with TNBC. Taken together, our findings suggest that MIR4500HG003 promotes TNBC metastasis through miR-483-3p-MMP9 axis that provides insight into potential prognostic markers for malignant TNBC.

## Results

### High expression of MIR4500HG003 enhances TNBC metastasis

**Fig. 1:**
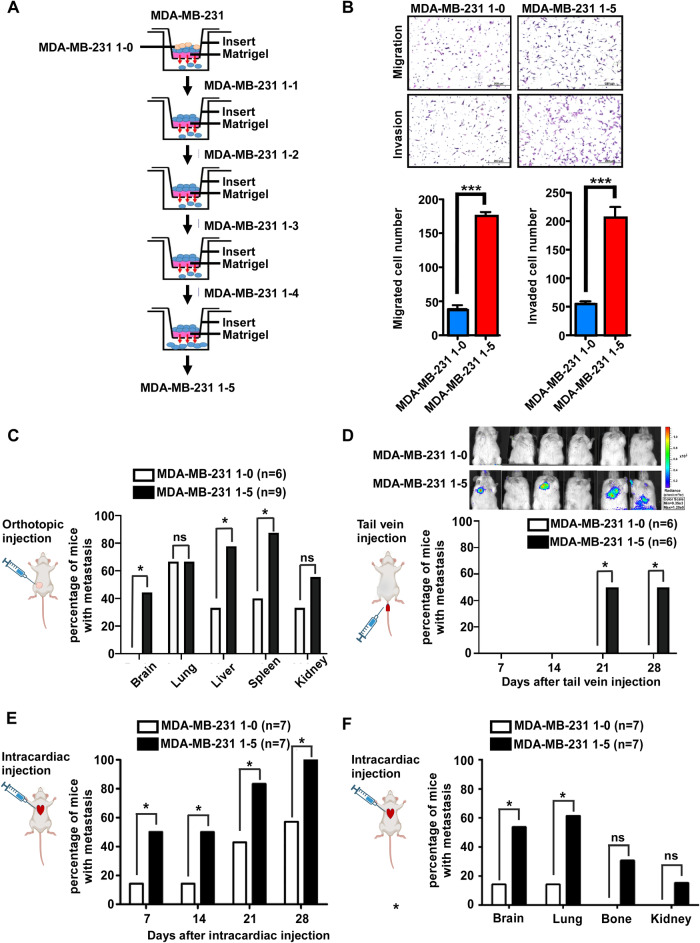
Establishion of highly migrating MDA-MB231. MDA-MB-231 1-5 cells exhibited higher abilities of metastasis in vitro and in vivo. **A** The selection process of highly invasive MDA-MB-231 subpopulation by transwell invasion assay. The in vitro selection was repeated five times sequentially to obtain highly metastatic MDA-MB-231 1-5 cells from parental MDA-MB-231 cells. The MDA-MB-231 1-0 cells were the cells remained on the top of the trans-well membrane. **B** MDA-MB-231 1-0 and MDA-MB-231 1-5 cells were used to examine migration and invasion abilities by transwell migration and invasion assays. **C** Fourteen weeks after orthotopic injection of MB231 1-0 and 1-5 cells, the incidence rate in specific distal organs was calculated. **D** Four weeks after tail vein injection of MB231 1-0 and 1-5 cells, the IVIS signal and metastasis incidence rate were calculated. **E**, **F** Four weeks after intracardiac injection of MB231 1-0 and 1-5 cells, the metastasis incidence rate and incidence rate in specific distal organs were calculated.

To identify the potential lncRNAs involved in TNBC metastasis, the highly metastatic TNBC cell lines derived from MDA-MB-231 cell lines were established from a series of in vitro selections (Fig. 1A). The un-invaded MDA-MB-231 cells were referred to as MDA-MB-231 1-0 cells (1-0), and the invaded MDA-MB-231 1-5 cells (1-5) were collected at fifth runs of in vitro invasion selection. The migrated and invaded cell numbers of 1-5 cells were significantly increased to 4.5 and 3.7-fold individually compared with 1-0 cells (Fig. 1B). The cell doubling time of 1-5 cells was 33.1 h whereas the 1-0 cells were 49.7 h (Supplementary Fig. 1A). In addition, the colony numbers of 1-5 cells were increased compared to the 1-0 by clonogenic assay (Supplementary Fig. 1B). These data suggested that 1-5 cells possessed high migration, invasion, and cell growth. To investigate whether 1-5 cells possess high metastasis in vivo, 1-0 and 1-5 cells with luciferase vectors were injected into female NOD/SCID mice, and luciferase-positive tumors in metastatic sites were monitored and analyzed as described in materials and methods [16, 17]. In the orthotopic injection model, the results of ex vivo showed that the metastases of organs were increased in 1-5 group when compared with 1-0 group (Fig. 1C and Supplementary Fig. 1C). In the tail-vein injection experiments, the 66.6% of mice had lung metastasis in 1-5 group whereas the metastatic rate was 0% in 1-0 cells at 4th week time point (Fig. 1D). In addition, in intracardiac injection model, the metastatic rate 50% of 1-5 group was higher than 14.2% of 1-0 group after 7days post-injection. After 28 days post-injection, the metastatic rate 100% of 1-5 group was higher than 57.1% of 1-0 group as well as high percentage of organ-specific metastasis when compared to the 1-0 group (Fig. 1E, F, Supplementary Fig. 1D). Taken together, MDA-MB-231 1-5 cells possess highly invasive and metastatic abilities in vitro and in vivo.

**Fig. 2:**
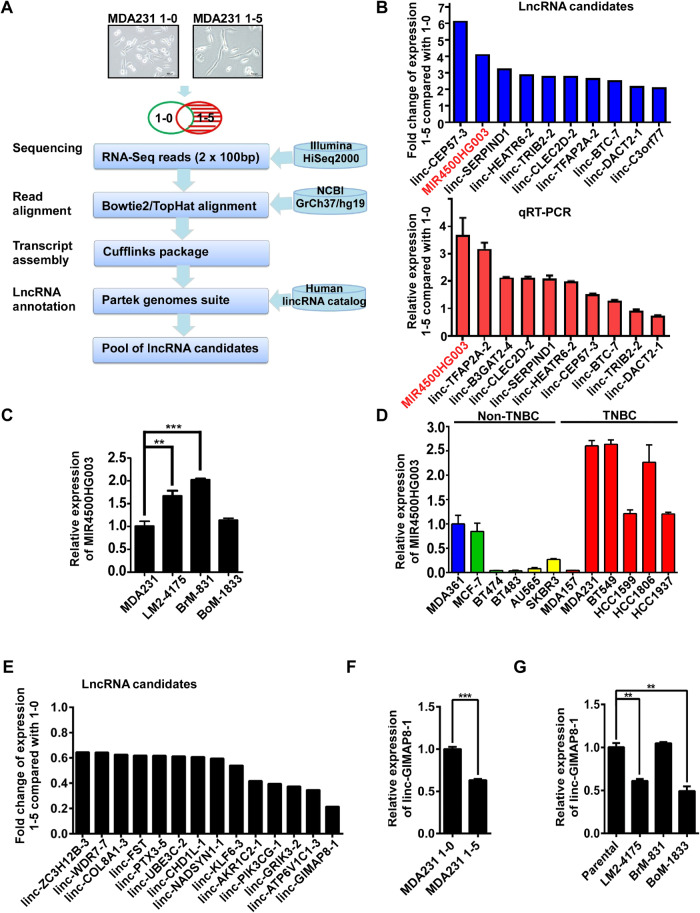
MIR4500HG003 is highly expression in highly migrating TNBC. **A** The flowcharts of RNA-seq and lncRNA annotation pipeline. Total RNA was extracted from MDA-MB-231 1-0 and MDA-MB-231 1-5 cells (in vitro selection). **B** The expression levels of lncRNA candidates in MDA-MB-231 1-5 cells were validated by qRT-PCR and the top 10 were shown. **C** The expression level of MIR4500HG003 was examined in MDA-MB-231 parental, LM2-4175(lung-tropic), BrM-831(brain-tropic), and BoM-1833(bone-tropic) by qRT-PCR analysis. **D** The expression level of MIR4500HG003 was examined in various BC cells by qRT-PCR analysis. **E** The downregulated candidate lncRNAs were shown in MDA-MB-231 1-5 cells. **F** The expression level of linc-GIMAP8-1 was examined in MDA-MB-231 1-0 and MDA-MB-231 1-5 cells. **G** The expression level of linc-GIMAP8-1 was examined in MDA-MB-231 parental and the derivatives by qRT-PCR analysis; **p < 0.01; ***p < 0.001.

To examine the different lincRNAs expression between highly and lowly metastatic TNBC cells, total RNAs from 1-5 and 1-0 cells were subjugated for RNA-Seq analysis. These sequencing reads were aligned to the NCBI GrCh37hg19 and the transcriptomes were assembled followed by lncRNA annotations with Human Body Map lincRNAs incorporation (Fig. 2A). To select upregulated lncRNAs in 1-5 cells for further investigation, the filtering criteria were set as below: (1) lncRNAs were identified without protein-coding potential by PhyloCSF score and the locus was conserved between human and mice by Ensembl Compara API; (2) differential expression of lncRNAs in 1-5 cells were greater than 2-fold when compared to 1-0; (3) the *p*-value of lncRNA between 1-5 and 1-0 cells was ≤0.01. The levels of top 10 lncRNAs candidates in 1-5 cells were 2-fold increase when compared to 1-0 and the validation of qRT-PCR showed that MIR4500HG003 was increased to nearly 4-fold in 1-5 cells (Fig. 2B). Interestingly, we found that MIR4500HG003 dominantly expressed in TNBC cells than non-TNBC cells (Fig. 2D). More Importantly, we found that MIR4500HG003 also was significantly upregulated in two highly metastatic organotropism TNBC cell lines, brain-metastatic BrM-831 and Lung-metastatic LM2-4175 cells (Fig. 2C), suggesting that MIR4500HG003 may play an important role in the metastasis of TNBC. Downregulated lncRNAs were also revealed as listed in Fig. 2E–G.

### High expression of MIR4500HG003 enhances TNBC migration and invasion

**Fig. 3:**
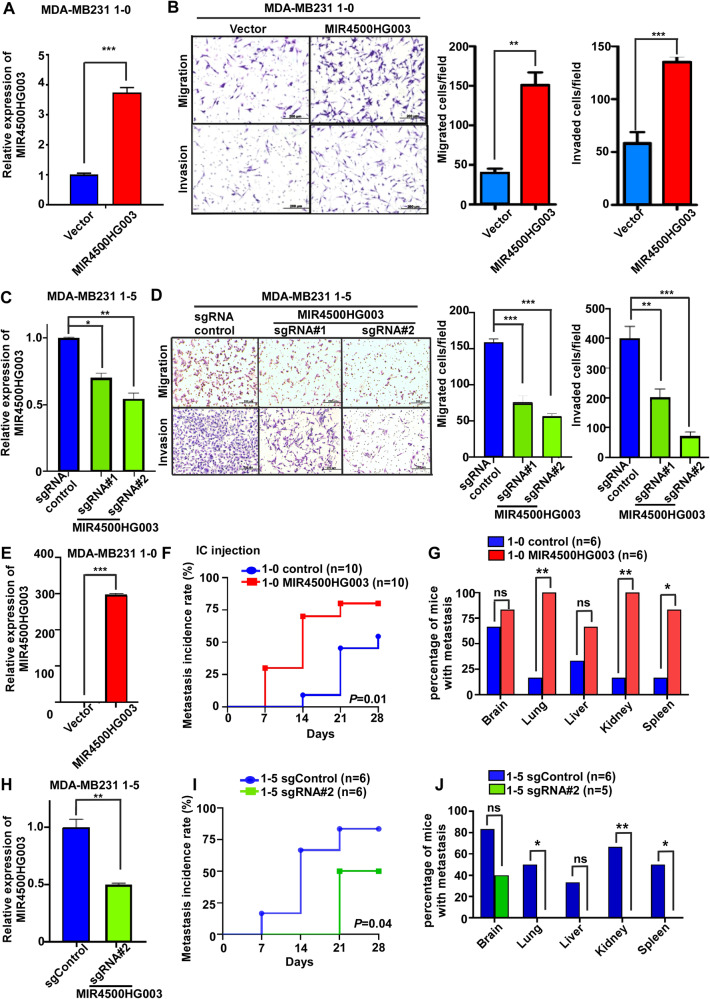
MIR4500-HG003 increases the abilities of metastasis in vitro and in vivo*.* **A** The expression level of MIR4500-HG003 was examined in MDA-MB-231 1-0 MIR4500-HG003 stable cells by qRT-PCR analysis. **B** MDA-MB-231 1-0 MIR4500-HG003 stable cells and control vector cells were used to examine migration and invasion abilities by transwell migration or invasion assay. **C** MDA-MB-231 1-5 cells were transiently transfected with siRNA targeting MIR4500-HG003 (50 nM). After 48 h post-transfection, the expression level of MIR4500-HG003 was examined by qRT-PCR analysis. **D** The migration and invasion abilities were examined by transwell migration or invasion assay. **E** The qRT-PCR was used to evaluate the MIR4500HG003 expression levels in MDA-MB-231 1-0 MIR4500HG003 stable cells. MDA-MB-231 1-0 MIR4500HG003 stable cells and control vector cells were inoculated into mice through intracardiac injection. The mice were exposed by IVIS weekly. **F** The percentage of mice with distant metastasis was calculated per week after intracardiac injection. **G** After 4 weeks post-injection, the IVIS signals were detected in various organs of mice and mice were sacrificed and the organs were collected for further analysis. **H** The qRT-PCR was used to evaluate the MIR4500HG003 expression levels in MDA-MB-231 1-5 sgMIR4500HG003 stable cells(sgRNA#2). sgRNA#2 and sgRNA control cells were inoculated into mice through intracardiac injection. The mice were exposed by IVIS weekly. **I** The percentage of mice with distant metastasis was calculated per week after intracardiac injection. **J** After 4 weeks post-injection, the IVIS signals were detected in various organs of mice, and mice were sacrificed and the organs were collected for further analysis; *p <0.05; **p <0.01; ***p <0.001.

To functionally characterize the role of MIR4500HG003 in TNBC metastasis, 1-0 cells with stable MIR4500HG003 overexpression (called 1-0 MIR4500HG003) or control vector (called 1-0 Vector) were generated. RT-PCR was performed to verify the ectopic expression of MIR4500HG003 after cell line generation. The expression of MIR4500HG003 was significantly increased to 3.7-fold (*p* < 0.001) in 1-0 MIR4500HG003 cells compared to 1-0 Vector cells (Fig. 3A). Next, under MIR4500HG003 overexpression condition, the migrated and invaded cells were significantly increased to 3.8 (*p* < 0.05) and 2.3-fold (*p* < 0.001), respectively in 1-0 MIR4500HG003 cells when compared with the 1-0 Vector cells (Fig. 3B). The similar phenomenon was also demonstrated in non-TNBC MCF7 overexpressing MIR4500HG003 stable cells (called MCF-7 MIR4500HG003). The migrated and invaded abilities were increases 1.5 and 2-fold individually in MCF-7 MIR4500HG003 cells when compared with MCF-7 vector cells (Supplementary Fig. 2A, B). To perform two-way demonstrations, two knockdown stable clones of 1-5 expressing MIR4500HG003 single guide RNA (sgRNA) (called 1-5 sgRNA#1 and 1-5 sgRNA#2) and sgRNA control vector (called 1-5 sgControl) were generated. The expression of MIR4500HG003 was significantly decreased to 70% (*p* < 0.05) in 1-5 sgRNA#1 and 46% (*p* < 0.01) in 1-5 sgRNA#2 compared to 1-5 sgControl cells (Fig. 3C). The migrated cells of 1-5 sgRNA#1 and sgRNA#2 cells were significantly decreased to 48% and 36% (*p* < 0.001) individually compared to 1-5 sgControl cells. The invaded cells of 1-5 sgRNA#1 and sgRNA#2 cells were significantly decreased to 50% (*p* < 0.01) and 18% (*p* < 0.001) individually compared to 1-5 sgControl cells (Fig. 3D). MIR4500HG003 small interfering RNAs (siRNAs) were also transiently transfected to decrease the MIR4500HG003 expression in 1-5 cells that the migration and invasion abilities were also inhibited (Supplementary Fig. 2C, D). Taken together, the above results suggested that MIR4500HG003 enhanced TNBC migration and invasion abilities. To examine the phenotype of MIR4500HG003 promotes TNBC metastasis in mice model, the overexpression of MIR4500HG003 in 1-0 MIR4500HG003 cells was validated by qRT-PCR and the organs were examined ex vivo for the presence of metastases after 4 weeks post-intracardiac injection (Fig. 3E). The results showed that the more IVIS signals of metastases in 1-0 MIR4500HG003 group compared to 1-0 vector group and the earlier metastasis incidence and higher percentage of metastasis in 1-0 MIR4500HG003 group (80%) compared to 1-0 Vector group (50%) within 4 weeks (*p* < 0.05, Fig. 3F and Supplementary Fig. 2E). Moreover, the results of ex vivo showed that the metastases of distal metastatic organs were increased in mice inoculated 1-0 MIR4500HG003 cells compared to 1-0 Vector cells (Fig. 3G and Supplementary Fig. 2F). In addition, the expression of MIR4500HG003 was significantly decreased to 46% (*p* < 0.01) in 1-5 sgRNA#2 cells compared to 1-5 sgControl cells (Fig. 3H). The IVIS signals of metastases were decreased in mice inoculated 1-5 sgRNA#2 cells compared to 1-5 sgControl cells and the later metastasis incidence and less percentage of metastases in 1-5 sgRNA#2 group (*p* < 0.02) compared to 1-5 sgControl group within 4 weeks (Fig. 3I and Supplementary Fig. 2G). Additionally, the results of ex vivo showed that the metastases of other organs were reduced in 1-5 sgRNA#2 group (*p* < 0.02) compared to 1-5 sgControl group (Fig. 3I and Supplementary Fig. 2H). In conclusion, these results suggested that MIR4500HG003 can promote metastatic abilities of TNBC cells in vitro and in vivo.

### MIR4500HG003 mediates the expression of MMP9 in TNBC cells

**Fig. 4:**
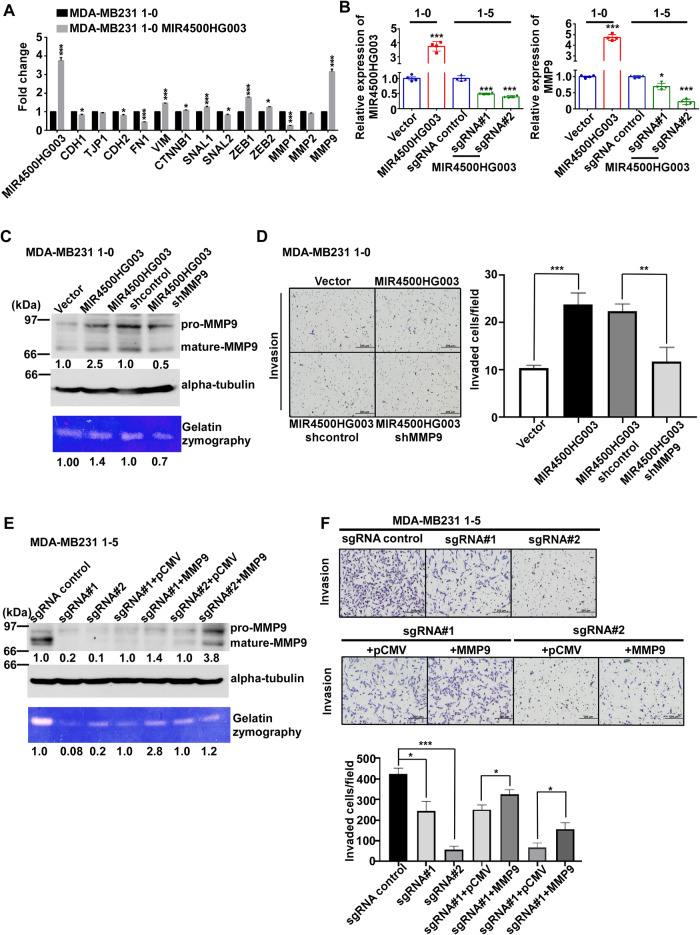
MIR4500-HG003 overexpression enhanced invasion through MMP9-dependent manner. **A** The expression level of EMT-related and MMP genes were examined by qRT-PCR analysis in MDA-MB-231 1-0 and MDA-MB-231 1-0 MIR4500HG003 stable cells. GAPDH was used as internal control. MIR4500HG003 stable cell lines and MIR4500HG003 knock-down stable cells were generated from MDA-MB-231 1-0 and MDA-MB-231 1-5 cells, respectively. **B** The expression of MIR4500HG003 and MMP9 were detected by qRT-PCR in these stable cell lines. **C** MDA-MB-231 1-0 vector and MIR4500HG003 stable cells were transfected with MMP9 shRNA or shControl followed by examining MMP9 protein level and enzymatic activity by Western blot analysis and gelatin zymography, respectively. α-tubulin was used as loading control. **D** MDA-MB-231 1-0 vector and MIR4500HG003 stable cells were transfected with MMP9 shRNA or shControl followed by examining cell invasive ability; ***p < 0.001. **E** MDA-MB-231 1-5 sgMIR4500HG003 stable cells and controls were overexpressed with MMP9 or control followed by examining MMP9 protein level and enzymatic activity by Western blot analysis and gelatin zymography, respectively. α-tubulin was used as loading control. **F** MDA-MB-231 1-5 sgMIR4500HG003 stable cells and controls were overexpressed with MMP9 or control followed by examining cell invasive ability; *p <0.05; **p <0.01; ***p <0.001.

The transition states of EMT play a significant role in tumor initiation, invasion, and metastasis. To investigate the molecular mechanism of MIR4500HG003-mediated TNBC metastasis, the total RNA of 1-0 MIR4500HG003 and vector cells was performed in qRT-PCR analysis to examine the EMT-related genes. The expressions of VIM, ZEB1, and MMP9 were significantly increased in 1-0 MIR4500HG003 cells compared to 1-0 vector cells (Fig. 4A). Moreover, the expression of MMP9 also was increased in 1-5 cells compared to 1-0 cells (Supplementary Fig. 3A). These results showed that MMP9 may act as a target protein of MIR4500HG003. To validate this hypothesis, we investigated the MMP9 expression in 1-0 MIR4500HG003, 1-5 sgRNA#1 and sgRNA#2 cells. The mRNA expression of MMP9 was significantly increased to 4.5-fold (p < 0.05) in 1-0 MIR4500HG003 cells and decreased to 70% and 20% in 1-5 sgRNA#1 and sgRNA#2 cells compared to control cells, respectively (Fig. 4B). Furthermore, the protein expression and enzyme activity of MMP9 were increased to 2.5 and 1.4-fold in 1-0 MIR4500HG003 cells compared to 1-0 vector cells (Fig. 4C). The protein expression and enzyme activity of MMP9 were decreased to 16% and 8% in 1-5 sgRNA#1 and 14% and 22% in 1-5 sgRNA#2 cells compared to 1-5 sgControl cells, respectively (Fig. 4E). To further investigate that MIR4500HG003 promotes TNBC metastasis thought MMP9, the knockdown of MMP9 were performed in 1-0 MIR4500HG003 cells and invasion assay was used to perform the metastasis ability of these cells. MMP9 protein expression and enzyme activity were decreased to 50% and 70% in 1-0 MIR4500HG003 cells transferred with pLKO.1-shMMP9 plasmid than shControl cells (Fig. 4C). The invaded cells in 1-0 MIR4500HG003 cells were significantly increased to 2.3-folds (*p* < 0.001) compared to 1-0 Vector group. When MMP9 was downregulated, the invaded cells in 1-0 MIR4500HG003+shMMP9 cells were significantly decreased to 50% (*p* < 0.01) than 1-0 MIR4500HG003 shControl cells (Fig. 4D). On the other hand, when MMP9 was overexpressed in MIR4500HG003 silenced 1-5 cells (Fig. 4E), the invaded cells were increased to 1.3 (*p* < 0.05) and 2.3-fold (*p* < 0.05) 1-5 sgRNA#1 + MMP9 and sgRNA#2 + MMP9 cells than control cells, respectively (Fig. 4F). These results indicated that MIR4500HG003 enhanced migration and invasion abilities of MDA-MB231 cells through MMP9-dependent mechanisms.

### MiR-483-3p physically interacts with MIR4500HG003

**Fig. 5:**
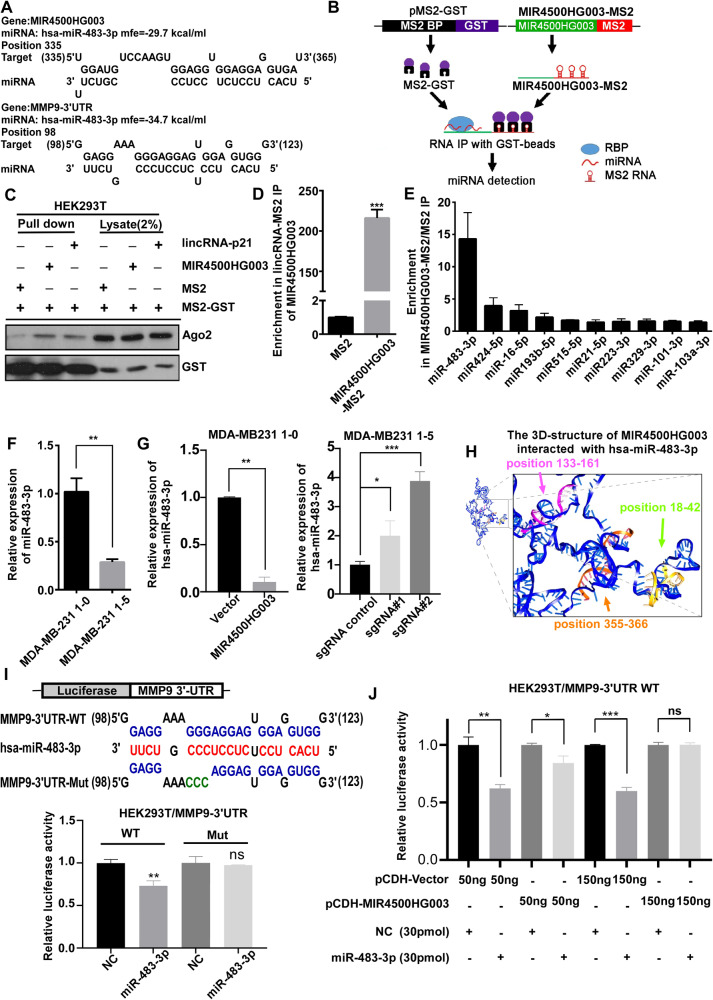
MIR4500-HG003 mediated MMP9 expression through miR-483-3p. **A** The $\Delta$G values of miR-483-3p interacting with MMP9-3’UTR or MIR4500-HG003 were calculated according to the sequencing alignment and predicted by RNAhybrid. **B** Schematic model of MS2-TRAP RIP system. **C** HEK293T was used in MS2-TRAP RIP assay and co-transfected with MIR4500HG003 tagged with MS2 RNA and MS2-GST. The primary antibodies against Ago2 and GST were used for Western Blot analysis. **D**, **E** The qRT-PCR was used to detect the expression level of MIR4500HG003 and the top 10 potential physically interacted microRNAs, which were predicted by bioinformatics. **F**, **G** The qRT-PCR was performed to examine the expression of miR-483-3p in MDA-MB231 1-0, 1-5, 1-0 MIR4500HG003 overexpression and 1-5 MIR4500HG003 knockdown groups. U6 miRNA is used as internal control. (*p <0.05; **p <0.01; ***p <0.001); **H** The 3D-structure of MIR4500HG003 was performed to build the tertiary structure of MIR4500HG003 and predict the binding sites of miR-483-3p on MIR4500HG003. **I** The sequencing alignment of miR-483-3p binding with MMP9-3’UTR WT or Mut. The binding activity for miR-483-3p onto MMP9-3’UTR was examined by luciferase reporter assay after transfection of miR-483-3p mimics (30 pmol). The relative luciferase activity in MMP9-3’UTR WT and mutant group was examined after 48 h of post transfection. **J** The binding activity for MIR4500HG003 interfered miR-483-3p onto MMP9-3’UTR was examined by luciferase reporter assay after co-transfection of miR-483-3p mimics (30 pmol) and MIR4500HG003 (50 ng, 150 ng). The relative luciferase activity in MMP9-3’UTR WT was examined after 48 h of post co-transfection.; *p <0.05; **p <0.01; ***p <0.001.

To answer how MIR4500HG003 regulates the MMP9 protein expression, the canonical pathways regulating the MMP9 expression was investigated, and the results showed that the upstream NF-κB and Erk pathways of MMP9 were unaffected in MDA231 1-0 MIR4500HG003 and MCF7 MIR4500HG003 cells (Supplementary Fig. 4A, B). Non-canonical pathways of MIR4500HG003 were then suggested to regulate MMP9 expression at the post-transcription level via unknown mechanism. According to cellular mechanism of lncRNA, it had been reported that miRNAs can act as mediators between lncRNAs and target proteins [16]. Bioinformatic analysis was performed to identify the miRNAs that can interact with both MIR4500HG003 and MMP9-3’UTR and the potential binding sites and calculated $\Delta$G were predicted by RNA-hybrid software (Fig. 5A). To investigate the miRNA-binding partners of MIR4500HG003, we used MS2-tagged RNA affinity purification (MS2-TRAP) assay to verify our potential miRNA candidates (Fig. 5B). The Ago2 expression was significantly increased in the pull-down lysate after overexpression of MIR4500HG003 tagged with MS2 RNA, suggesting that MIR4500HG003 can interact with miRNA in the RNA-induced silencing complex (RISC) (Figs. 5C,D). Here we used the levels of lincRNA-p21 and let-7b-5p as positive controls [16] in the pull-down lysate after MIR4500HG003 overexpression (Supplementary Fig. 4C), indicating that MIR4500HG003 can interact with miRNAs in the RISC. Figure 5E illustrates that miR-483-3p was the most prominent among the identified miRNAs, displaying a substantial 14.3-fold enrichment in the MIR4500HG003-MS2 pull-down lysate compared to MS2 RNA only (refer to Supplementary Fig. 4D). Furthermore, the results from qRT-PCR showed that the expression of miR-483-3p was decreased to 28% (*p* < 0.001) in 1-5 cells compared with 1-0 cells (Fig. 5F). Meanwhile, the expression of miR-483-3p was decreased to 10% (*p* < 0.01) in 1-0 MIR4500HG003 cells compared to 1-0 Vector cells and increased to 1.8 (*p* < 0.05) and 3.9-fold (*p* < 0.001) in 1-5 sgRNA#1 and sgRNA#2 cells compared to 1-5 sgControl cells (Fig. 5G). Moreover, a possible three-dimensional (3D) structure of MIR4500HG003 bound with miR-483-3p at three sites was predicted (Fig. 5H). RNAhybrid was applied to identify and rank potential miR-483-3p binding sites on MIR4500HG003, and three top-ranked sites (position: 335, 18, and 133 with predicted binding free energy −29.7, −22.9, and −18.7 kcal/ml, respectively) were considered to build a model structure of MIR4500HG003 simultaneously bound with three miR-483-3p sequences (Supplementary Fig. 4E, F). These results suggested that MIR4500HG003 interacted with miR-483-3p.

### MIR4500HG003 regulates the expression of MMP9 through competitively sponging miR-483-3p, which can bind on 3’UTR of MMP9 mRNA

To investigate whether miR-483-3p can regulate MMP9 expression directly at the post-transcriptional level, we generated the MMP9 3’-UTR constructs of wild type (WT) and mutant (Mut, seed region Δ106–108 nt, Fig. 5I) and performed luciferase reporter assay. The luciferase activity was dose-dependent decreased under the different concentrations of miR-483-3p in the WT group (Supplementary Fig. 4G). Figure 5I found that under 30 pmol miR-483-3p condition, the luciferase activity was 17% decreased (*p* < 0.01) in the WT group, but unaffected in the mutant group, suggesting that miR483-3p can interact with MMP9 3’-UTR through direct binding to the region of 98–124 nt. To demonstrate miR483-3p is the shared miRNA for MIR4500HG003 and MMP9, transfection of either miR483-3p mimics alone or in combination with MIR4500HG003 overexpression in reporter assay was performed. Figure 5J showed that under 30 pmol miR483-3p condition, the luciferase activity was decreased to 62% (*p* < 0.01) but the activities were restored to 84% (*p* < 0.05) or unaffected after dual overexpression of miR483-3p and MIR4500HG003. In addition, we investigated the potential direct interaction between MIR4500HG003 and MMP9 using the MS2-TRAP assay. The results revealed no detectable expression of either MMP9 RNA or protein in the MIR4500HG003-MS2 pull-down lysate (Supplementary Fig. 4H). Collectively, these findings suggest that MIR4500HG003 may exert an inhibitory effect on miR483-3p-mediated MMP9 expression.

**Fig. 6:**
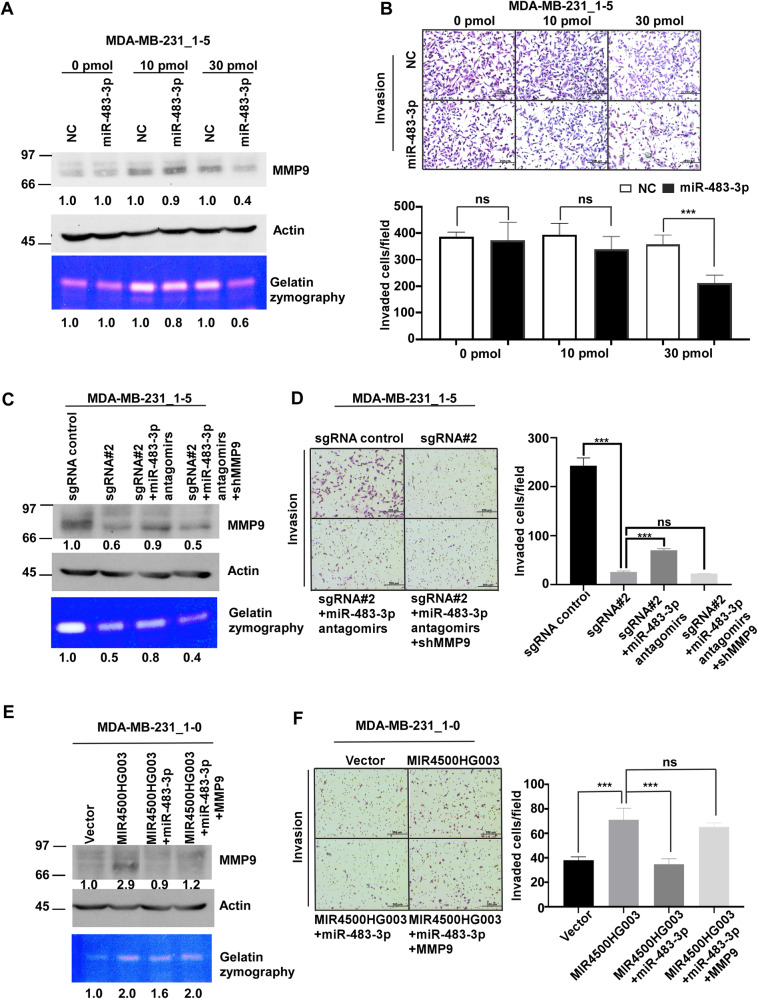
miR-483-3p partially abolished MIR4500-HG003 mediated MMP9 effect in TNBC cells. **A** MDA-MB-231 1-5 cells were transfected with different dose miR-483-3p mimics followed by examining MMP9 protein level and enzymatic activity by Western blot analysis and gelatin zymography, respectively. Actin was used as loading control. **B** MDA-MB-231 1-5 cells were transfected with different dose miR-483-3p mimics followed by examining cell invasive ability; ***p < 0.001. **C** MDA-MB-231 1-5 sgMIR4500HG003 stable cells and controls were co-transfected with miR-483-3p antigomirs/shControl or miR-483-3p antigomirs/shMMP9 followed by examining MMP9 protein level and enzymatic activity by Western blot analysis and gelatin zymography, respectively. Actin was used as loading control. **D** The ability of cell invasion was examined by transwell assay. **E** MDA-MB-231 1-0 MIR4500HG003 stable cells and controls were co-transfected with miR-483-3p mimics/pCMV or miR-483-3p mimics/MMP9 followed by examining MMP9 protein level and enzymatic activity by Western blot analysis and gelatin zymography, respectively. Actin was used as loading control. **F** The ability of cell invasion was examined by transwell assay.

To clarify the role of miR483-3p in TNBC metastasis, 1-5 cells were transfected with miR-483-3p mimics. The results of Fig. 6A showed that the protein levels and enzyme activity of MMP9 were does-dependent decreased under different miR-483-3p conditions. Moreover, the invaded cells were decreased to 82% and 57% (*p* < 0.001) under 10 and 30 pmol of miR-483-3p mimics condition (Fig. 6B). Furthermore, the migrated cells were also decreased under the same condition (Supplementary Fig. 5A). These results demonstrated that the restoration of miR-483-3p can down-regulate the expression of MMP9 in dose-dependent and inhibit migration and invasion abilities of MDA-MB231 cells in vitro. To further investigate whether MIR4500HG003 can promote TNBC metastasis through miR-483-3p-MMP9 axis, we performed the migration and invasion assays in 1-5 sgRNA#2 cells with miR-483-3p antagomirs overexpression under the condition of MMP9 knockdown. Figure 6C showed that the protein level and enzyme activity of MMP9 in 1-5 sgRNA#2 group were decreased to 60% and 50% of control group. In contrast, the protein level and enzyme activity of MMP9 in 1-5 sgRNA#2 under 30 pmol miR-483-3p antagomirs condition were restored to 90% and 80% of control cells. However, the protein level and enzyme activity of MMP9 in 1-5 sgRNA#2 under miR-483-3p antagomirs and shMMP9 overexpression condition were decreased to 50% and 40% compared to control cells. In invasion assay, the invaded cells in 1-5 sgRNA#2 cells were decreased to 11% (*p* < 0.001) of 1-5 sgControl cells. In contrast, the invaded cells in 1-5 sgRNA#2 cells under miR-483-3p antagomirs overexpression condition were increased to 2.7-fold (*p* < 0.001) of 1-5 sgRNA#2 cells. Furthermore, the invaded cells of 1-5 sgRNA#2 cells transferred with miR-483-3p antagomirs and shMMP9 were decreased to 33% of 1-5 sgRNA#2 cells transferred with miR-483-3p antagomirs only (Fig. 6D). Moreover, the migration abilities of 1-5 sgRNA#2 were increased under miR-483-3p antagomirs expression condition and decreased following shMMP9 expression (Supplementary Fig. 5B). On the other hands, the protein level and enzyme activity of MMP9 were increased to 2.9 and 2.0-fold in 1-0 MIR4500HG003 compared to 1-0 vector cells. However, the protein level and enzyme activity of MMP9 were changed to 0.9 and 1.6-fold in 1-0 MIR4500HG003 under miR-483-3p mimics expression compared to 1-0 vector cells. Additionally, the protein level and enzyme activity of MMP9 were increased to 1.2 and 2.0-folds in 1-0 MIR4500HG003 under miR-483-3p mimics and MMP9 expression compared to 1-0 vector cells (Fig. 6E). In invasion assay, the invaded cells in 1-0 MIR4500HG003 cells were increased to 1.9-folds (*p* < 0.001) compared to 1-0 Vector cells. In contrast, the invaded cells in 1-0 MIR4500HG003 cells under miR-483-3p mimics expression were decreased to 49% (*p* < 0.001) compared to 1-0 MIR4500HG003 cells. Furthermore, the invaded cells were increased to 1.9-fold in 1-0 MIR4500HG003 cells under miR-483-3p mimics and MMP9 expression condition compared to miR-483-3p mimics expressing 1-0 MIR4500HG003 cells (Fig. 6F). Similarly, the migration abilities of 1-0 MIR4500HG003 cells were decreased under miR-483-3p mimics expression condition. However, the migrated cells didn’t much alter in 1-0 MIR4500HG003 cells under miR-483-3p mimics and MMP9 expression (Supplementary Fig. 5C). To investigate the in vivo role of miR-483-3p in metastasis, we utilized 1-5 cells transfected with negative control miRNAs (1-5 NC) and 1-5 cells transfected with miR-483-3p mimics (1-5 miR-483-3p). Subsequently, these cells were IC injected into NOD/SCID female mice, and metastatic presence was assessed weekly using IVIS imaging. Organs were examined ex vivo 4 weeks post-intracardiac injection. Results indicated a higher IVIS signal of metastases in the 1-5 NC group compared to the 1-5 miR-483-3p group. Metastasis incidence was earlier, and the photon flux was slightly higher in the 1-5 NC group within 4 weeks (Supplementary Fig. 5D). Ex vivo findings revealed increased metastases in distal organs, particularly in the brain (*p* < 0.05) and liver (*p* < 0.05), in mice inoculated with the 1-5 NC group compared to the 1-5 miR-483-3p group (Supplementary Fig. 5E). Taken together, miR-483-3p/MMP9 axis plays important role in mediating migration and invasion abilities in TNBC.

### High expression levels of MIR4500HG003 are correlated with tumor recurrence in TNBC patients

**Fig. 7:**
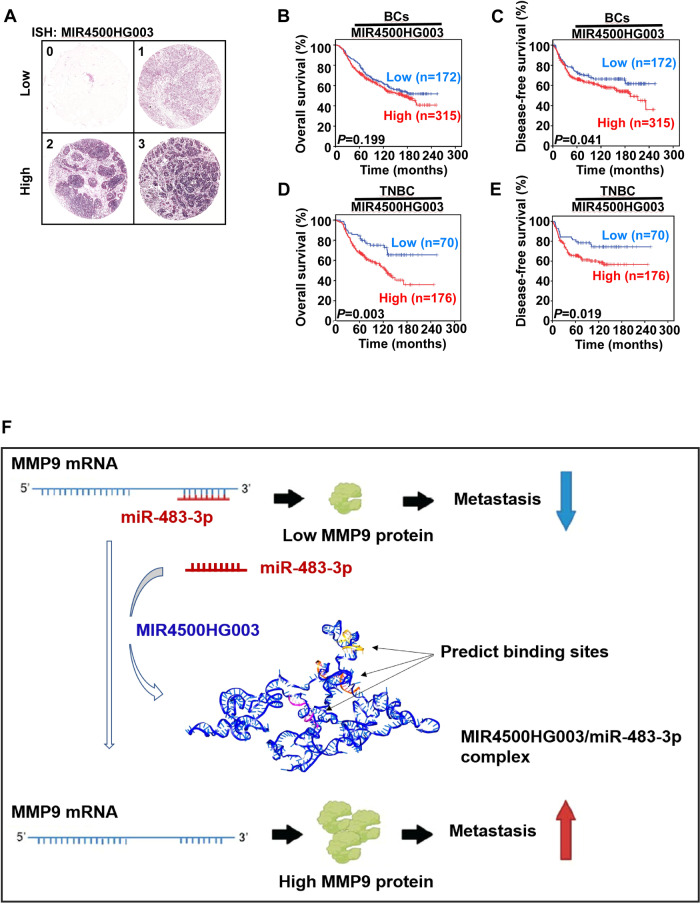
High level of MIR4500HG003 was correlated with tumor recurrence in TNBC patients. **A** Representative in situ hybridization images of breast cancer specimens showing MIR4500HG003 expression level scored from 0 to 3. The score 0 and 1 were classified to low MIR4500HG003 expression. The score 2 and 3 were defined to high MIR4500HG003 expression. **B** The overall survival and (**C**) disease-free survival of 487 breast cancer patients were stratified with MIR4500HG003 expression level by Kaplan-Meier analysis. **D** The overall survival and (**E**) disease-free survival of 246 TNBC were stratified with MIR4500HG003 expression level by Kaplan-Meier analysis.; **p < 0.01; ***p < 0.001. **F** Schematic model showed how MIR4500HG003 mediated TNBC metastasis.

To investigate the relationship between disease progress and MIR4500HG003 expression in BC patients, we analyzed a BC tissue microarray that contained 488 BC specimens collected from KVGH and conducted an in situ hybridization assay to evaluate the expression of MIR4500HG003. Each sample was scored from 0–3 according to MIR4500HG003 expression levels and then subdivided into the MIR4500HG003 Low (0, 1) and High groups (2, 3) (Fig. 7A). Kaplan-Meier analysis was used to investigate the associations between MIR4500HG003 expression levels and overall survival (OS) and disease-free survival (DFS). Our results indicated that the BC patients with high expression of MIR4500HG003 were significantly correlated with poorer DFS (*p* = 0.04, Fig. 7B) but not OS (*p* = 0.2, Fig. 7C). The TNBC patients with high expression of MIR4500HG003 had poorer OS (*p* = 0.003, Fig. 7D) and DFS (*p* = 0.02, Fig. 7E). Chi-squared analysis was used to investigate the relationships between various clinicopathological characteristics of the BC patients and their MIR4500HG003 expression levels. The results showed that high MIR4500HG003 expression was significantly associated with recurrence in BC (*p* = 0.04) and TNBC (*p* = 0.01) patients (Table 1 and Table 2). These results indicated that MIR4500HG003 was correlated with poor OS, DFS, and metastasis in TNBC patients.

**Table 1:** MIR4500HG003 vs BC patient characteristics.

BC	MIR4500HG003			
	488	173	315	
	N	Low	High	*P* value
Age				
<45	143	56	87	0.27
>45	345	117	228	
Stage				
I–II	341	120	221	0.8548
III–IV	147	53	94	
Survival status				
Yes	221	76	145	0.6335
No	266	97	169	
Recurrence status				
Yes	190	59	131	0.1048
No	298	114	184	
Metastasis				
Yes	188	56	132	**0.0384**
No	300	117	183	
Skin and lymph Node				
Yes	66	17	49	0.0767
No	422	156	266	
Supraclavicular lymph Node				
Yes	36	7	29	**0.037**
No	452	166	286	
Breast				
Yes	17	4	13	0.2956
No	471	169	302	
Lung and Pleura				
Yes	94	27	67	0.1292
No	394	146	248	
Liver				
Yes	56	22	34	0.5237
No	432	151	281	
Bone				
Yes	83	30	53	0.8847
No	405	143	262	
Brain				
Yes	29	7	22	0.1891
No	459	166	293	
Chest				
Yes	2	1	1	0.6665
No	486	172	314	
Adrenal Gland				
Yes	0	0	0	N/A
No	488	173	315	

Bold values indicates statistically significant *p* values less than 0.05.

**Table 2:** MIR4500HG003 vs TNBC patient characteristics.

TNBC	MIR4500HG003			
	246	70	176	
	N	Low	High	Chi-test (*P* value)
Age				
<45	65	23	42	0.1489
>45	181	47	134	
Stage				
I–II	178	56	122	0.091
III–IV	68	14	54	
Survival status				
Yes	103	21	82	**0.0173**
No	143	49	94	
Recurrence status				
Yes	84	17	67	**0.0397**
No	162	53	109	
Metastasis				
Yes	85	16	69	**0.015**
No	161	54	107	
Skin and lymph Node				
Yes	34	6	28	0.1324
No	212	64	148	
Supraclavicular lymph Node				
Yes	16	2	14	0.1435
No	230	68	162	
Breast				
Yes	7	1	6	0.3993
No	239	69	170	
Lung and Pleura				
Yes	47	8	39	0.0534
No	199	62	137	
Liver				
Yes	16	4	12	0.7514
No	230	66	164	
Bone				
Yes	28	5	23	0.1867
No	218	65	153	
Brain				
Yes	14	2	12	0.2263
No	232	68	164	
Chest				
Yes	2	1	1	0.4977
No	244	69	175	
Adrenal Gland				
Yes	0	0	0	N/A
No	246	70	176	

Bold values indicates statistically significant *p* values less than 0.05.

## Discussion

In the last decade, lncRNAs have been reported to participate in the metastatic cascade and found to correlate with clinical outcomes [18, 19]. In this study, we identified a novel lncRNA, MIR4500HG003, which plays important role in TNBC metastasis through MMP9. Overexpression of MIR4500HG003 in MDA-MB-231 cells enhanced cell metastasis in vitro and in vivo. The results of MS2-TRAP and MMP9-3’UTR reporter assay showed that MIR4500HG003 can enhance the stability of MMP9 mRNA by competitively sponging miR-483-3p and further increase expression of MMP9. Orthotopic injection model demonstrates the entire process of tumor metastasis. Cancer cells were injected into the tail vein or ventricles of mice as a metastasis model from survival in the circulation to metastatic colonization [20]. Three independent human metastatic animal models demonstrated the high metastasis ability of cells with high MIR4500HG003 expression. Our findings highlight on the underlying molecular mechanisms and reveal the interaction between MIR4500HG003 and miR-483-3p, as well as the regulation of MMP9 (Fig. 7F). The clinical relevance of MIR4500HG003 is evident, as TNBC patients with high MIR4500HG003 expression have significantly poorer OS and DFS. This suggests that MIR4500HG003 may serve as a diagnostic and prognostic marker for TNBC patients to predict and prevent severe distant metastasis, ultimately improving patient outcomes. This study suggests that MIR4500HG003 can promote TNBC metastasis through MIR4500HG003/miR-483-3p/MMP9 axis and acts as a potential therapeutic target and prognostic biomarker for TNBC patients.

## Materials and methods

### Cell lines

Human breast cancer cells (BCCs) Human BCCs MDA-MB-361 (ATCC Cat# HTB-27; RRID: CVCL_0620), MCF7 (ATCC Cat# HTB-22; RRID: CVCL_0031), BT474 (ATCC Cat# HTB-20; RRID: CVCL_0179), BT483 (ATCC Cat# HTB-121; RRID: CVCL_2319), AU565 (ATCC Cat# CRL-2351; RRID: CVCL_1074), SKBR3 (ATCC Cat# HTB-30; RRID: CVCL_0033), MDA-MB-157 (ATCC Cat# HTB-24, RRID: CVCL_0618), MDA-MB-231 (ATCC Cat# HTB-26, RRID: CVCL_0062), BT549 (ATCC Cat# HTB-122, RRID: CVCL_1092), HCC1599 (ATCC Cat# CRL-2331, RRID: CVCL_1256), HCC1806 (ATCC Cat# *CRL-2335*, *RRID*: CVCL_ 1258), and HCC1937 (ATCC Cat# CRL-2336; RRID: CVCL_0290) were cultured in the growth medium supplemented with 10% fetal calf serum (FCS) and 1% penicillin-streptomycin (P/S). MDA-MB-231 containing luciferase were obtained from Dr. Michael Hsiao’s Lab (Genomics Research Center, Academia Sinica, Taipei, Taiwan). MDA-MB-231 parental and its derivatives LM2-4175 [21], BrM-831 [22] and BoM-1833 [23] were kindly gifts from Dr. Tang-Long Shen’s Lab (National Taiwan University, Taipei, Taiwan) and cultured in Dulbecco’s Modified Eagle Medium (DMEM), supplemented with 10% fetal calf serum (FCS), and 1% penicillin-streptomycin (P/S). All cell lines were maintained at 37 °C in a humidified atmosphere of 5% CO_2_.

### Clinical specimens

Human breast cancer tissue microarrays TMA-BCs, including two clinical cohorts of patients, were created from KVGH (Kaohsiung Veterans General Hospital) archives and clinical tissues were collected with informed consent and with approval from Institutional Reviewed Board approval (VEGHKS12-CT9-07 and VEGHKS13-CT11-18). All tissue sections with tumor parts were fixed by 10% formalin, dehydrated, and paraffin-embedded and further histologically examined for the presence of tumor with hematoxylin and eosin (HE) and immunohistochemistry stain.

### Stable cell line generation

The full-length cDNA of MIR4500HG003 was amplified by PCR with the primers: forward (5′-CGGAATTCCTCGTGTACTTACTGTTAAGGATC-3′) and reverse (5′-AAATATGCGGCCGCTCTATATCAATATTAAACATATGATT-3′) and constructed into pCDH Expression Lentivector (System Biosciences, Palo Alto, CA, USA) for the establishment of MDA-MB-231 MIR4500HG003 stable cell lines. The sequences for MIR4500HG003 knockdown were constructed into pAll-dCas9-KRAB.pPuro (RNAi Core Facility, Academic Sinica) for the establishment of MDA-MB-231 sg MIR4500HG003 stable cell lines. Next, Lentivirus carrying sequences for MIR4500HG003 overexpression or knockdown were generated by co-transfection with packaging plasmid (psPAX2) and envelope plasmid (pMD.2G) into HEK293T. After 48 h post-transfection, the supernatant containing viral particles was harvested and filtered via 0.45 μm filters. Lentiviral infection was performed and stable clones were selected by puromycin and enriched by fluorescence-activated cell sorting (FACS).

### RNA sequencing (RNA-Seq) and lncRNA annotation pipeline

The process of RNA-Seq was used the platform Illumina HiSeq2000^™^ (National Center for Genome Medicine, Taipei, Taiwan). Briefly, the rRNA-depleted sample preparation was needed before the construction of cDNA library. The sequencing was performed on a single lane of a flow cell, sequenced as 2 × 100 bp following the manufacturer’s instructions. Next, the sequencing reads were aligned to NCBI GrCh37hg19 using Bowtie2/TopHat [24, 25], followed by transcriptome assembly using Cufflinks package [26]. LncRNA annotation was analyzed by Partek Genomic Suite 6.6 with incorporation of Human Body Map lincRNAs [27]. Our candidates of lncRNAs were eventually validated by qRT-PCR analysis.

### RNA extraction, cDNA synthesis and quantitative real-time PCR (qRT-PCR)

Total RNAs from human BCCs were extracted by Zymo Direct-zolRNA MiniPrep Kit (Zymo Research, Irvine, CA, USA) and stored at −80 °C freezer. The cDNA synthesis for lncRNAs and miRNAs was performed by M-MLV reverse transcriptase system kit (Life Technologies, Carlsbad, CA, USA) according to the manufacturer’s instruction and stored at −20 °C for further studies. The cDNAs from human BCCs and KAPA SYBR® FAST Master Mix (2X) Universal (Kapa Biosystems, Wilmington, MA, USA) were used in the reaction by Applied Biosystems StepOne^™^ to examine expression levels of target genes. All reactions were performed in triplicate and relative gene expressions were calculated by 2^−ΔΔCT^ method. *GAPDH* or *RNU6B* was used as internal control.

### Migration and invasion assays

MDA-MB-231 cells (5 × 10^5^ cells/ml) were seeded in 8 mm-pore size inserts (BD Biosciences, Bedford, MA, USA) coated with BD Matrigel Matrix (BD Biosciences). Serum-free medium and growth medium containing FCS were added into each insert and incubated at 37 °C for 8 h. Invasive cells (INV) were trypsinized from the bottom of the membrane and cultured in a 6-well plate with growth medium. The in-vitro selection was repeated 5 times sequentially to obtain MDA-MB-231 1-5. To select MDA-MB-231 1-0, the same procedure was used and the cells remained on the top of the membrane were collected. MDA-MB-231 1-0 and 1-5 cells (1 × 105 cells/ml) were seeded in 8 mm-pore size inserts (BD Biosciences, Bedford, MA, USA). Serum-free and growth medium containing FCS were added into the upper and lower layer of each insert. For migration assay, cells were incubated at 37 °C for 5 h. For invasion assay, the inserts were coated with 1 mg/ml of Matrigel Matrix (BD Biosciences) and incubated at 37 °C for 17 h. Subsequently, the lower side of cells were fixed with methanol and stained with 10% Giemsa (Sigma). The images were captured under the microscopy Olympus IX71 and the cells were counted by ImageJ.

### Western blot analysis

Protein lysates from human BCCs were loaded on SDS-polyacrylamide gel for electrophoresis and transferred to PVDF membranes. Protein expressions were examined after incubation with primary antibodies followed by HRP-conjugated secondary antibodies and ECL-enhanced chemiluminescence solution (PerkinElmer). Primary antibodies MMP9 (ab76003, Abcam), Ago2 (ab186733, Abcam), GST (sc-138 Santa Cruz Biotechnology), β-actin (A5441, Sigma) and α-tubulin (T5168, Sigma) were used. The blot intensity was quantified by ImageJ.

### Gelatin zymography

The conditioned media of human BCCs were centrifuged at 3500 rpm for 10 min and collected. The concentrated media were loaded on 10% SDS-polyacrylamide gel for electrophoresis containing 0.1% of gelatin (Sigma). After electrophoresis, the gelatin gels were washed with 2.5% Triton X-100 at RT for 1.5 h to remove SDS followed by incubation with incubation buffer (50 mM Tris-HCl, 0.15 M NaCl, and 10 mM CaCl_2_) at 37 °C for 48 h. The gels were stained with 0.05% Coomassie Blue (Sigma) and washed with destaining buffer. The gelatinase activity of MMP9 was determined as clear bands against the background of undigested bands and the images were captured by the scanner.

### In vivo metastasis assay

For mice experiments, we established orthotopic, tail-vein, and intracardiac injection model. All animal experiment protocols were approved by the Institutional Animal Care and Use Committee (IACUC) in NCKU. All animal experiment protocols were approved by the Institutional Animal Care and Use Committee (IACUC) in NCKU. To establish an orthotopic BC tumor model, NOD/SCID female mice of age 4–6 weeks were selected for xenografting studies. 1 × 10^5^ of cells were used to be counted and orthotopically injected with Matrigel (9.7 mg/ml, BS Biosciences) into mice. The growth of primary tumor was monitored by IVIS system once a week. The primary tumor and metastatic organs were surgically removed and measured the levels of luciferase activity after ~14 weeks. For tail vein and intracardiac injection, 1 × 10^5^ cells were injected to tail vein or the lateral ventricle per mouse, respectively. Mice bearing tumors were imaged by IVIS 200 Imaging System (PerkinElmer) every week and the signal were also analyzed by Living ImageR 4.0 software. The metastatic organs were surgically removed and measured the levels of luciferase activity after ~4 weeks.

### In situ hybridization (ISH)

IsHyb In Situ Hybridization Kit (BioChain, Newark, CA, USA) was used according to the manufacturer’s instructions. Briefly, paraffin-embedded xenograft tissues and human TMA were deparaffinized and rehydrated followed by 4% paraformaldehyde fixation in 1X DEPC-PBS. Slides were treated 10 μg/ml of proteinase K (Biochain) and incubated with pre-hybridization solution at 50 °C for 4 h before hybridization with Custom LNA^™^ lncRNA Probes (Exiqon, Vedbaek, Denmark) at 45 °C overnight. Then slides were stringently washed with SSC buffer followed by AP-conjugated anti-digoxingenin antibody incubation at RT for 4 h. The signals were eventually visualized by NBT/BCIP staining and counterstained with Nuclear Fast Red. The intensity of MIR4500HG003 expressions was quantified by HistoQuest Cell Analysis.

### MS2-tagged RNA affinity purification (MS2-TRAP)

HEK293T was transiently co-transfected with 2 μg of pMS2-MIR4500HG003 or pMS2 plus 1 μg of pMS2-GST. After 48 h post-transfection, cells were lysed in 300 μl of lysis buffer (20 mM Tris-HCl at pH7.5, 100 mM KCl, 5 mM MgCl_2_, 10 mM DTT, 0.5% NP-40, protease inhibitors and RNase inhibitor) and incubated the supernatant with 50 μl of GSH agarose beads slurry at 4 °C for 4 h. Then the supernatant were washed with NT2 buffer (50 mM Tris-HCl at pH7.5, 150 mM NaCl, 1 mM MgCl_2_ and 0.05% NP-40) [28]. For Western blot analysis, the primary antibody Ago2 from Abcam and GST from Santa Cruz Biotechnology were used. For qRT-PCR assay, the supernatant was incubated with 0.1% SDS and 0.5 mg/ml Proteinase K before RNA purification. The expression levels of lncRNAs and miRNAs were further validated.

### Simulation of 3D modeling

RNAhybrid (https://bibiserv.cebitec.uni-bielefeld.de/rnahybrid) was applied to identify and rank potential miR-483-3p binding sites on MIR4500HG003, and three top-ranked sites (position 335, position 18, and position 133 with predicted binding free energy −29.7 kcal/ml, −22.9 kcal/ml, and −18.7 kcal/ml, respectively) were considered to build a model structure of MIR4500HG003 simultaneously bound with three miR-483-3p sequences. 3dRNA was utilized to construct an initial MIR4500HG003 structure where we extracted the segments at position 335, position 18, and position 133 and manually threat with removal of their intramolecular hybridization and local minimization to smoothen the conformation. Three miR-483-3p sequences to hybridize the segments were sequentially added at the three positions followed by local structural optimization using the steepest descent algorithm and conjugate gradient algorithm. A short duration with 1 ns equilibration and 20 ns production of molecular dynamics simulations using AMBER18 program [29] was carried out. The entire complex structure was immersed in a TIP3P water box in which the distance separating any RNA atom from the water box wall was managed at least 10 Å. In addition to 0.15 M NaCl for physiological conditions, the water box also contained 674 sodium cations for neutrality of the studied RNA complex. A cutoff of 8 Å was applied to treat nonbonding interactions, the particle mesh Ewald method [30] was used to treat long-range electrostatic interactions, and the SHAKE algorithm [31, 32] was applied to constrain all hydrogen bonds.

### Luciferase reporter assay

The full-length sequence of MMP9 3’UTR was amplified by PCR using the primer set: MMP9 3’UTR F: 5′-ACTAGTGGCTCCCGTCCTGCTTTG-3′ and R: 5′-AAGCTTCTGACCTCGTGATCCGCC-3′ followed by cloned into pMIR-REPORT Luciferase Vector (Thermo Fisher Scientific, Waltham, MA, USA). The mutant form of MMP9 3’UTR was constructed by two-step site-directed mutagenesis. For reporter assay, cells were co-transfected with wild-type or mutant of MMP9 3’UTR and Renilla luciferase as reference control. After 48 h post-transfection, reporter assay was performed by Dual-Luciferase Reporter Assay system (Promega, Madison, WI, USA).

### Statistical analysis

All the statistical analyses were performed by Prism6 and SPSS Statistics 20.0 (IBM Corporation, Armonk, NY, USA). The survival curves were analyzed by Kaplan-Meier method and compared with a log-rank test. Data from all three independent experiments were presented as the mean ± SD and analyzed by unpaired two-tailed Student’s *t* test. For all tests, *p* < 0.05 was used to define statistical significance (*p <0.05, **p <0.01, ***p <0.001, ****p <0.0001).

### Supplementary information

Supplemental information

MIR4500HG003 3D-structure prediction movie

Raw data of Western blotting

## Data Availability

All data generated or analyzed during this study are included in the published article and its supplementary information files.
