# Supplementary material for: A novel long non-coding RNA MIR4500HG003 promotes tumor metastasis through miR-483-3p-MMP9 axis in triple-negative breast cancer
Source: Cell Death Dis. 2024 May 2;15(5):310. doi: 10.1038/s41419-024-06675-w (PMC11065892; doi:10.1038/s41419-024-06675-w)
Supplement: Supplementary file 1 — Supplemental information [file 41419_2024_6675_MOESM1_ESM.docx]

**Supplemental information**

**
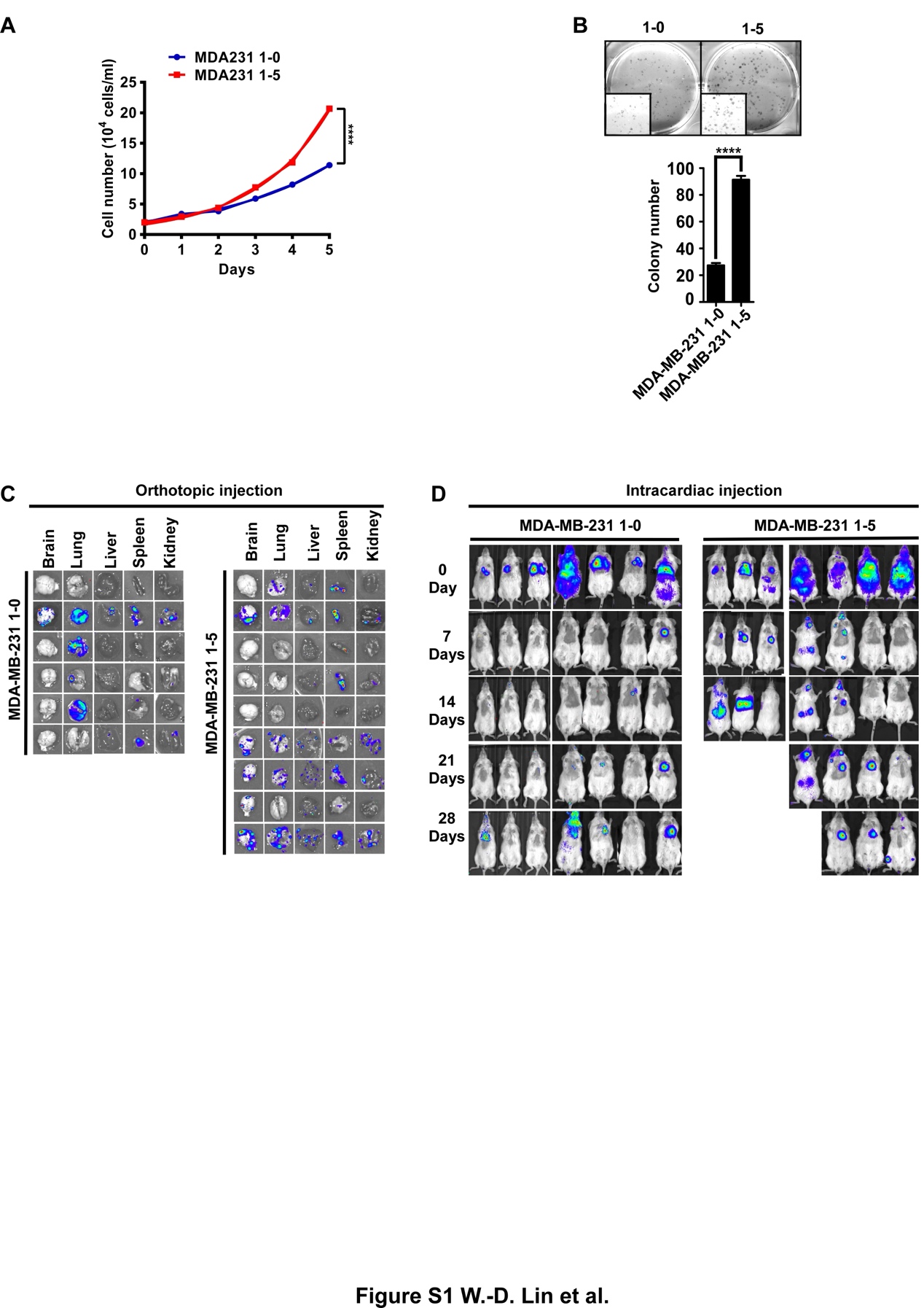
**

**Figure S1.** MDA-MB-231 1-5 cells exhibited high proliferative ability. MDA-MB-231 1-0 and MDA-MB-231 1-5 cells were used to examine (A) cell proliferative activity and anchorage-independent growth ability by (B) clonogenic assay; **p* < 0.05; *****p* < 0.0001. (C) MB231 1-0 and 1-5 cells were fat-pad injected into NOD-SCID mice. After 14 weeks, the organs were collected and detected through IVIS system *ex vivo*.

(D) Four weeks after intracardiac injection of MB231 1-0 and 1-5 cells, the IVIS signals of metastatic cells were used to calculate the percentage of mice in distant metastasis once a week.

**
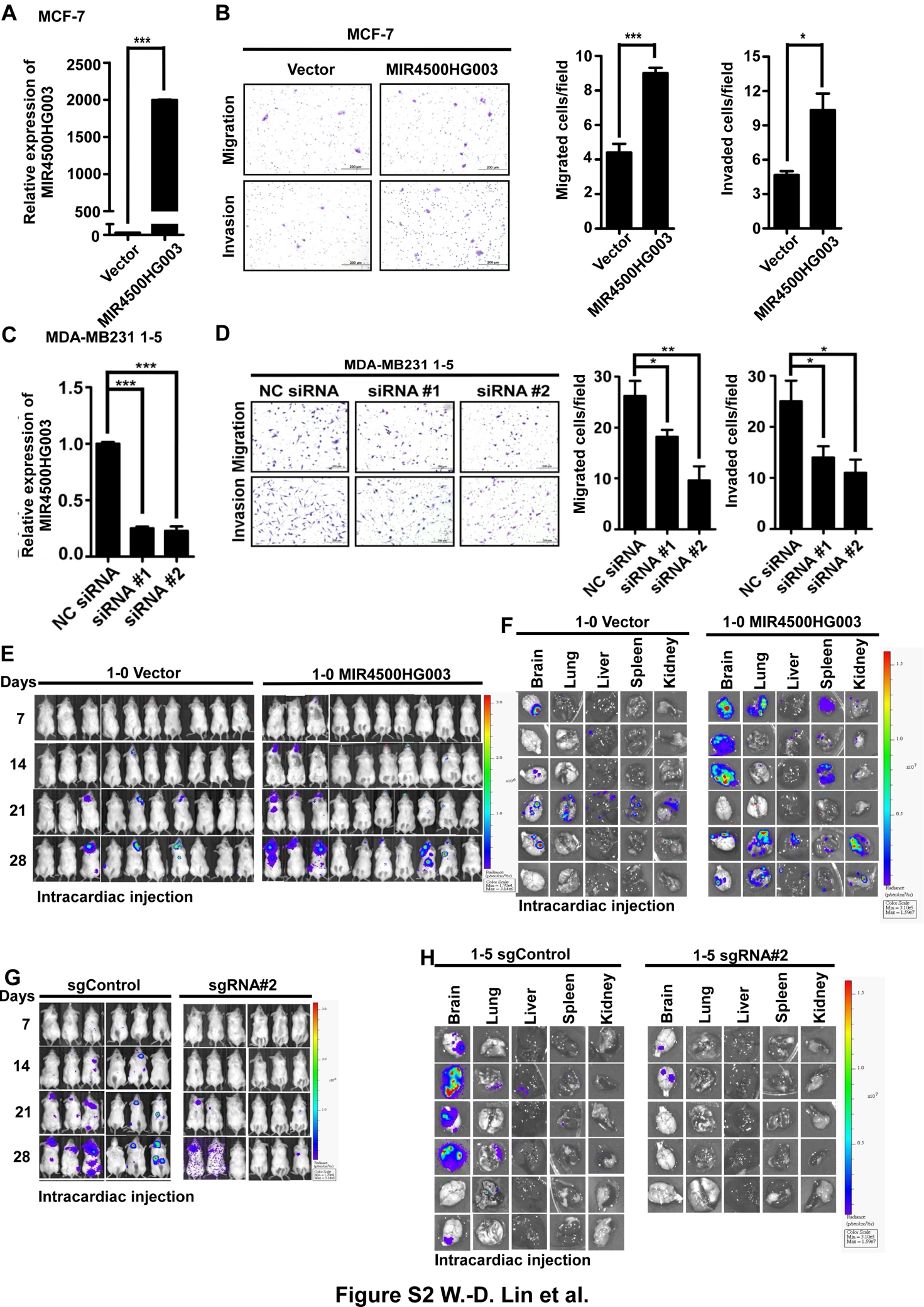
Figure S2.** MIR4500-HG003 increases the abilities of metastasis *in vitro* and *in vivo*

(A) The expression level of MIR4500-HG003 was examined in MIR4500-HG003 overexpressing MCF-7 cells by qRT-PCR analysis. (B) MIR4500-HG003 overexpressing MCF-7 cells cells and control vector cells were used to examine migration and invasion abilities by transwell migration or invasion assay. (C) MDA-MB-231 1-5 cells were transiently transfected with siRNA targeting MIR4500-HG003 (50 nM). After 48 h post-transfection, the expression level of MIR4500-HG003 was examined by qRT-PCR analysis; ***p < 0.001. (D) The migration and invasion abilities were examined by transwell migration or invasion assay; * p < 0.05; **p < 0.01.

(E) MDA-MB-231_1-0 MIR4500HG003 stable cells and control vector cells were inoculated into mice through intracardiac injection. The mice were exposed by IVIS weekly. The IVIS signals of mice with distant metastatic cells was calculated per week after intracardiac injection. (F) After 4 weeks post-injection, the organs were collected and detected through IVIS system *ex vivo*. (G) sgRNA#2 and sgRNA control cells were inoculated into mice through intracardiac injection. The mice were exposed by IVIS weekly. The IVIS signals of mice with distant metastatic cells was calculated per week after intracardiac injection. (H) After 4 weeks post-injection, the organs were collected and detected through IVIS system *ex vivo*; * *p* < 0.05; ***p* < 0.01; ****p* < 0.001.

**
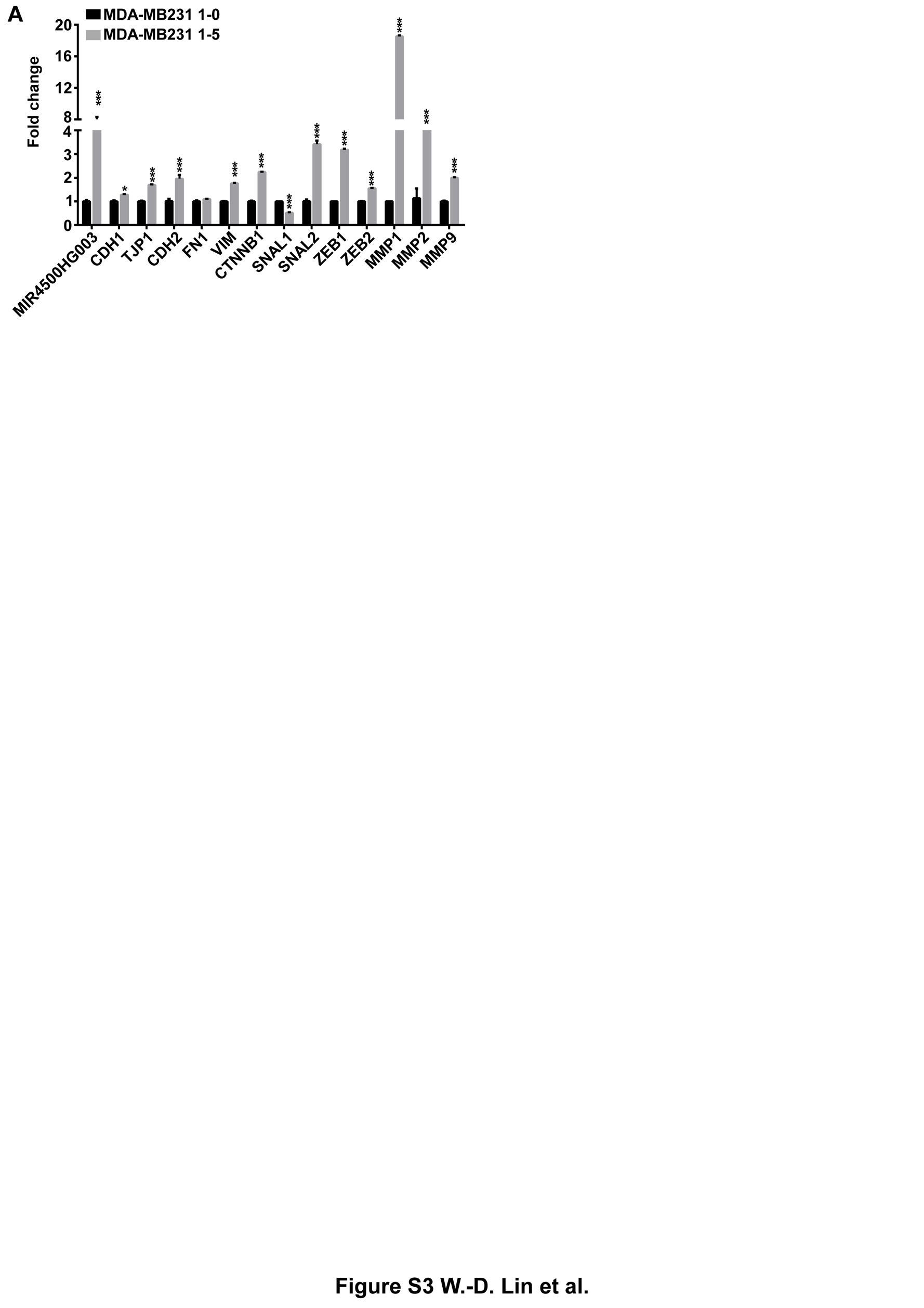
**

**Figure S3. (A)** The qRT-PCR was used to examine the expression level of EMT-related and MMP genes in MDA-MB-231 1-0 and 1-5 cells.

**
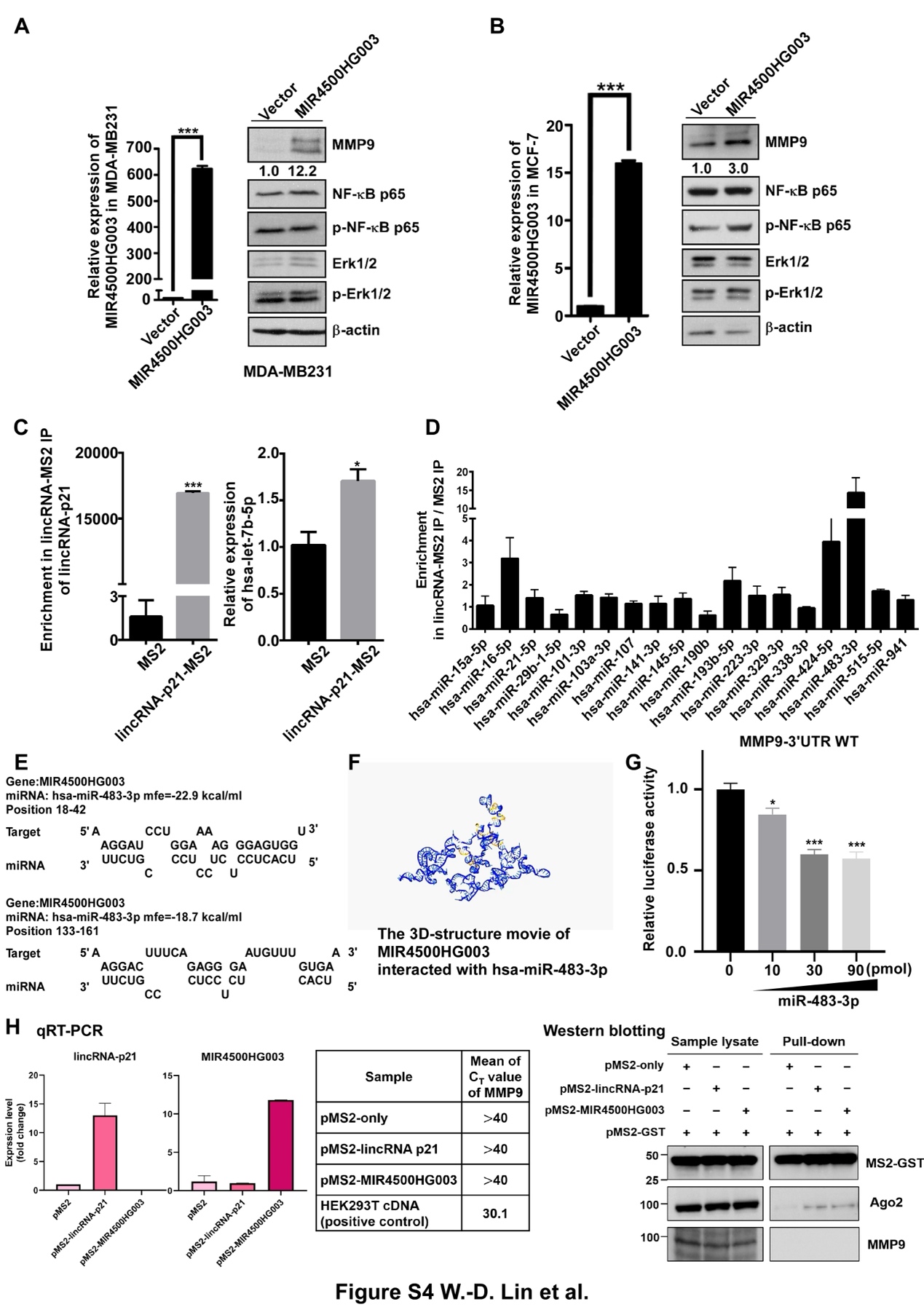
**

**Figure S4.** (A) MIR4500HG003 overexpression enhanced MMP9 level in MDA-MB-231 cells. The expression level of MIR4500HG003 and MMP9 were examined by qRT-PCR analysis in MDA-MB-231 vector and MIR4500HG003 stable cells. 18S rRNA was used as internal control. The protein levels of MMP9, NF-ĸβ, p-NF-ĸβ, Erk1/2, p-Erk1/2, E-cadherin and vimentin were examined by Western blot analysis. β-actin were used as loading controls; ****p* < 0.001. (B) MIR4500HG003 overexpression enhanced MMP9 level in MCF-7 cells. The expression level of MIR4500HG003 and MMP9 were examined by qRT-PCR analysis in MCF-7 vector and MIR4500HG003 stable cells. 18S rRNA was used as internal control. The protein levels of MMP9, NF-ĸβ, p-NF-ĸβ, Erk1/2, p-Erk1/2, E-cadherin and vimentin were examined by Western blot analysis. β-actin were used as loading controls; ****p* < 0.001.(C) HEK293T was used in MS2-TRAP RIP assay and co-transfected with MIR4500HG003 tagged with MS2 RNA and MS2-GST. The physical interaction between lincRNA-p21 and hsa-let7b-5p are demonstrated in previous study and used as positive control in MS2-TRAP RIP system. The expression level of lincRNA-p21 and hsa-let-7b-5p were examined by qRT-PCR. RUN6B was used as internal control. (D) The qRT-PCR was used to detect the expression level of potential physically interacted microRNAs, which were predicted by bioinformatics. (E) The △G values of miR-483-3p interacting with MIR4500-HG003 were calculated according to the sequencing alignment and predicted by RNAhybrid. (F)The 3D-structure movies of MIR4500HG003 interacted with miR-483-3p. (G)The binding activity for miR-483-3p onto MMP9-3’UTR was examined by luciferase reporter assay after transfection of miR-483-3p mimics (0 to 90 pmol). The relative luciferase activity in MMP9-3’UTR WT and mutant group was examined after 48 h of post transfection. (H) HEK293T was used in MS2-TRAP RIP assay and co-transfected with MIR4500HG003 tagged with MS2 RNA and MS2-GST. The expression level of lincRNA-p21, MIR4500HG003 and MMP9 were examined by qRT-PCR. RUN6B was used as internal control. The primary antibodies against GST, Ago2, and MMP9 were used for Western Blot analysis.


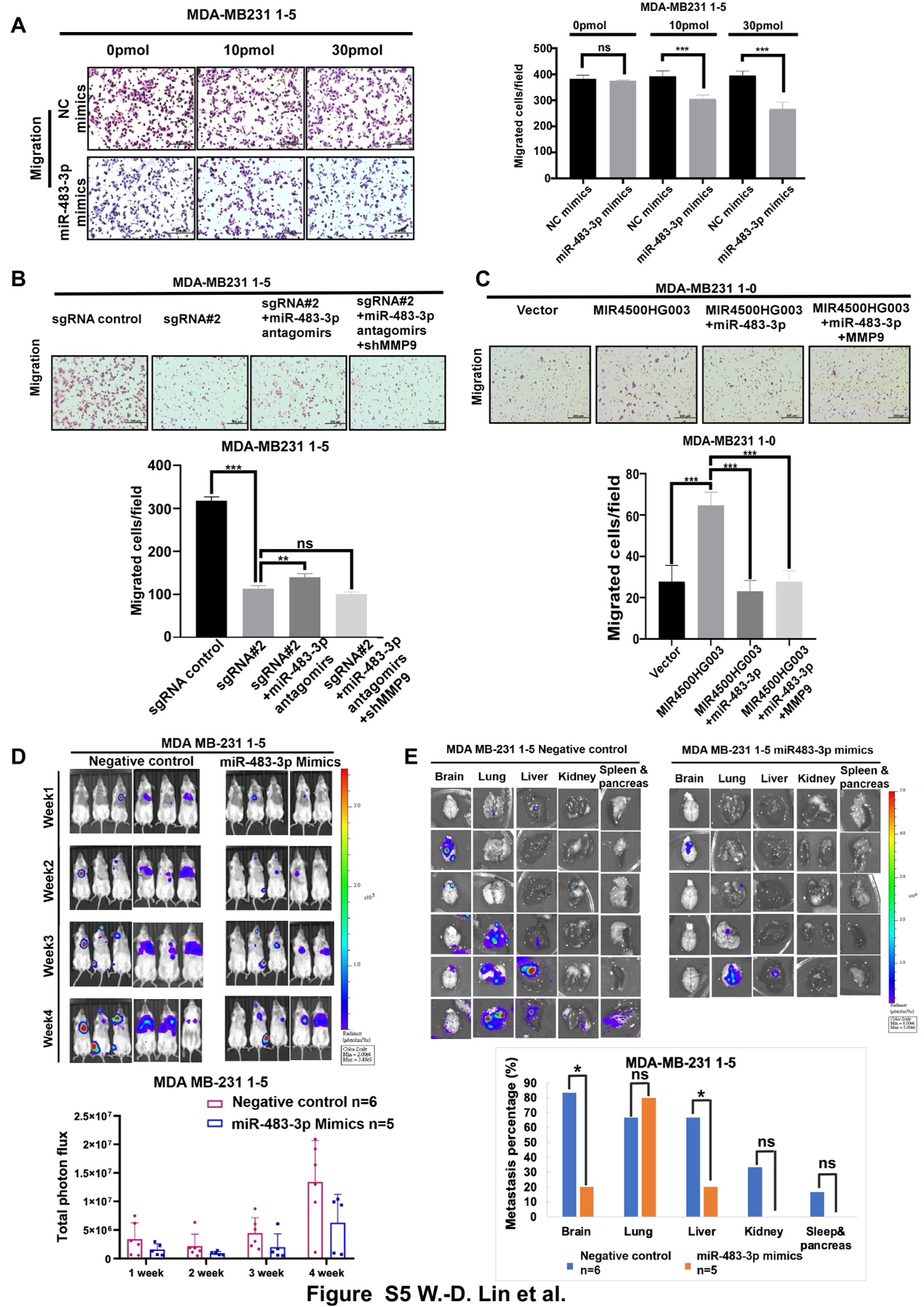


**Figure S5.** (A)MiR-483-3p mimics decrease the migration ability of MDA-MB231 1-5 cells in dose-dependent. Migration assay was performed to examine the migration ability of cells. The migrated cells are decreased in miR-483-3p mimics manipulation 1-5 cells. (B)MiR-483-3p antagomirs abolished the decrease of migration ability caused by sgMIR4500HG003. Migration assay was performed to examine the migration ability of cells. The migrated cells were increased in 1-5 sgRNA#2 cells after miR-483-3p antagomirs manipulation. (C) Migration assay was performed to examine the migration ability of cells. The migrated cells were decreased in 1-0 MIR4500HG003 overexpression cells after miR-483-3p mimics manipulation. (D) MDA-MB-231 1-5 cells transiently transferred with negative control miRNAs or 30 pmol miR-483-3p mimics were inoculated into mice through intracardiac injection. The mice were exposed by IVIS weekly. The IVIS signals of mice with distant metastatic cells was calculated per week after intracardiac injection. (E) After 4 weeks post-injection, the organs were collected and detected through IVIS system *ex vivo*. (* p < 0.05; ** p < 0.01; *** p < 0.001; ns, not significant difference)

**RT-PCR primer pair for lncRNA genes:**

| **Primer name** | **Sequences (5' to 3')** |
| --- | --- |
| MIR4500HG003 Forward | GTT CCT CCT AGC TGC AAA CA |
| MIR4500HG003 Reverse | TTC CCT TGT GTT GCC TAG AC |
| linc-TFAP2A-2 Forward | GGA GTC TGT GGC AAA AGT CA |
| linc-TFAP2A-2 Reverse | CGC AGG TTA GGT ACG CTT AA |
| linc-B3GAT2-4 Forward | ACA CAA CAC AGG AGG GGA TA |
| linc-B3GAT2-4 Reverse | CCT TTC CCT CTG CTC AAC AA |
| linc-CLEC2D-2 Forward | GTT CTG GGA GAT GAC CAA TG |
| linc-CLEC2D-2 Reverse | GCA AAC AGG ACC ATA TCA GG |
| linc-SERPIND1 Forward | AAT TGG TGA CGC TGC AGA AT |
| linc-SERPIND1 Reverse | CGG TCT GCT CCT GGA ATA TG |
| linc-HEATR6-2 Forward | ATG AGT GTG TTG CAG GAT GA |
| linc-HEATR6-2 Reverse | AAG GGA TGA CGA TCT TAG GG |
| linc-CEP57-3 Forward | ACT CTC TTT TGG GGC TGT TG |
| linc-CEP57-3 Reverse | ACC GCG AGT CCT AGT GAA AA |
| linc-BTC-7 Forward | TCC TGC TTC CCT ATC TTC AA |
| linc-BTC-7 Reverse | TCC ATC CAC AGT TCA GAG GT |
| linc-TRIB2-2 Forward | CGA GTC ATG CAC ACT TCA TC |
| linc-TRIB2-2 Reverse | ATG CTC TGC ACC TGT ATG GT |
| linc-DACT2-1 Forward | CTC CTC CAT CCA ACA ACA AG |
| linc-DACT2-1 Reverse | GGG GTT CTT CCA AGG TCT TA |
| lincRNA-p21 Forward | CCT GTC CAC TCG CTT TC |
| lincRNA-p21 Reverse | GGA ACT GGA GAC GGA ATG TC |

**RT-PCR primer pair for miRNA genes:**

| **Primer name** | **Sequences (5' to 3')** |
| --- | --- |
| hsa-let-7b-5p RT | GTC GTA TCC AGT GCA GGG TCC GAG GTA TTC GCA CTG GAT ACG ACA ACC AC |
| hsa-let-7b-5p Forward | CGG TGA GGT AGT AGG TTG TGT GGT T |
| hsa-miR-15a-5p RT | GTC GTA TCC AGT GCA GGG TCC GAG GTA TTC GCA CTG GAT ACG ACC ACA AA |
| hsa-miR-15a-5p Forward | CCG GCG GTA GCA GCA CAT AAT G |
| hsa-miR-16-5p RT | GTC GTA TCC AGT GCA GGG TCC GAG GTA TTC GCA CTG GAT ACG ACC GCC AA |
| hsa-miR-16-5p Forward | CGG CGG CTA GCA GCA CGT AAA T |
| hsa-miR-21-5p RT | GTC GTA TCC AGT GCA GGG TCC GAG GTA TTC GCA CTG GAT ACG ACT CAA CA |
| hsa-miR-21-5p Forward | CGG CGG CTA GCT TAT CAG ACT G |
| hsa-miR-22-3p RT | GTC GTA TCC AGT GCA GGG TCC GAG GTA TTC GCA CTG GAT ACG ACA CAG TT |
| hsa-miR-22-3p Forward | CGG CGG AAG CTG CCA GTT GAA |
| hsa-miR-29b-1-5p RT | GTC GTA TCC AGT GCA GGG TCC GAG GTA TTC GCA CTG GAT ACG ACT CTA AA |
| hsa-miR-29b-1-5p Forward | CGG CGG GCT GGT TTC ATA TGG |
| hsa-miR-101-3p RT | GTC GTA TCC AGT GCA GGG TCC GAG GTA TTC GCA CTG GAT ACG ACT TCA GT |
| hsa-miR-101-3p Forward | CGG CGG CGT ACA GTA CTG TGA TA |
| hsa-miR-103a-3p RT | GTC GTA TCC AGT GCA GGG TCC GAG GTA TTC GCA CTG GAT ACG ACT CAT AG |
| hsa-miR-103a-3p Forward | CGG CGG AGC AGC ATT GTA CAG |
| hsa-miR-107 RT | GTC GTA TCC AGT GCA GGG TCC GAG GTA TTC GCA CTG GAT ACG ACT GAT AG |
| hsa-miR-107 Forward | CGG CGG AGC AGC ATT GTA CAG |
| hsa-miR-141-3p RT | GTC GTA TCC AGT GCA GGG TCC GAG GTA TTC GCA CTG GAT ACG ACC CAT CT |
| hsa-miR-141-3p Forward | CCG GCG GTA ACA CTG TCT GGT A |
| hsa-miR-145-5p RT | GTC GTA TCC AGT GCA GGG TCC GAG GTA TTC GCA CTG GAT ACG ACA GGG AT |
| hsa-miR-145-5p Forward | CGG CGA GTC CAG TTT TCC CAG |
| hsa-miR-190b RT | GTC GTA TCC AGT GCA GGG TCC GAG GTA TTC GCA CTG GAT ACG ACA ACC CA |
| hsa-miR-190b Forward | CGG CGG CGG ATG ATA TGT TTG ATA T |
| hsa-miR-193b-5p RT | GTC GTA TCC AGT GCA GGG TCC GAG GTA TTC GCA CTG GAT ACG ACT CAT CT |
| hsa-miR-193b-5p Forward | CGG ATA CGG GGT TTT GAG GGC |
| hsa-miR-203a-3p RT | GTC GTA TCC AGT GCA GGG TCC GAG GTA TTC GCA CTG GAT ACG ACC TAG TG |
| hsa-miR-203a-3p Forward | GCC GGC GGG TGA AAT GTT TAG GA |
| hsa-miR-204-5p RT | GTC GTA TCC AGT GCA GGG TCC GAG GTA TTC GCA CTG GAT ACG ACA GGC AT |
| hsa-miR-204-5p Forward | CGG CGG TTC CCT TTG TCA TCC |
| hsa-miR-223-3p RT | GTC GTA TCC AGT GCA GGG TCC GAG GTA TTC GCA CTG GAT ACG ACT GGG GT |
| hsa-miR-223-3p Forward | CCC GGC GGT GTC AGT TTG TCA AA |
| hsa-miR-329-3p RT | GTC GTA TCC AGT GCA GGG TCC GAG GTA TTC GCA CTG GAT ACG ACA AAG AG |
| hsa-miR-329-3p Forward | CGG CGG CAA CAC ACC TGG TTA A |
| hsa-miR-338-3p RT | GTC GTA TCC AGT GCA GGG TCC GAG GTA TTC GCA CTG GAT ACG ACC AAC AA |
| hsa-miR-338-3p Forward | CGG CGG TCC AGC ATC AGT GAT |
| hsa-miR-424-5p RT | GTC GTA TCC AGT GCA GGG TCC GAG GTA TTC GCA CTG GAT ACG ACT TCA AA |
| hsa-miR-424-5p Forward | CGG CGG CAG CAG CAA TTC ATG |
| hsa-miR-483-3p RT | GTC GTA TCC AGT GCA GGG TCC GAG GTA TTC GCA CTG GAT ACG ACA AGA CG |
| hsa-miR-483-3p Forward | CGG CAT TCA CTC CTC TCC TCC |
| hsa-miR-515-5p RT | GTC GTA TCC AGT GCA GGG TCC GAG GTA TTC GCA CTG GAT ACG ACC AGA AA |
| hsa-miR-515-5p Forward | GGC GGC GGT TCT CCA AAA GAA AG |
| hsa-miR-941 RT | GTC GTA TCC AGT GCA GGG TCC GAG GTA TTC GCA CTG GAT ACG ACG CAC AT |
| hsa-miR-941 Forward | AAT TGG CAC CCG GCT GTG TGC |
| RNU6B Forward | GCT TCG GCA GCA CAT ATA CTA |
| RNU6B Reverse | AAA ATA TGG AAC GCT TCA CGA |
| Universal reverse primer | CCA GTG CAG GGT CCG AGG TAT |

**RT-PCR primer pair for protein-coding genes and reference controls:**

| **Primer name** | **Sequences (5' to 3')** |
| --- | --- |
| CDH1 Forward | GAA AGC GGC TGA TAC TGA CC |
| CDH1 Reverse | CGT ACA TGT CAG CCG CTT C |
| TJP1 Forward | GTG TTG TGG ATA CCT TGT |
| TJP1 Reverse | GAT GAT GCC TCG TTC TAC |
| CDH2 Forward | TGT TTG ACT ATG AAG GCA GTG G |
| CDH2 Reverse | TCA GTC ATC ACC TCC ACC AT |
| VIM Forward | CTC TTC CAA ACT TTT CCT CCC |
| VIM Reverse | AGT TTC GTT GAT AAC CTG TCC |
| FN1 Forward | ACC TGT ACA CCT TGA ATG ACA |
| FN1 Reverse | TGA TAC CAG CAA GGA ATT GGG |
| CTNNB1 Forward | AAA ATG GCA GTG CGT TTA G |
| CTNNB1 Reverse | TTT GAA GGC AGT CTG TCG TA |
| SNAI1 Forward | ACC ACT ATG CCG CGC TCT T |
| SNAI1 Reverse | GGT CGT AGG GCT GCT GGA A |
| SNAI2 Forward | TTC GGA ACA CAT TAC CT |
| SNAI2 Reverse | ATG GGT CTG CAG ATG AGC CC |
| ZEB1 Forward | TTC AAA CCC ATA GTG GTT GCT |
| ZEB1 Reverse | TGG GAG ATA CCA AAC CAA CTG |
| MMP1 Forward | AGC TAG CTC AGG ATG ACA TTG ATG |
| MMP1 Reverse | GCC GAT GGG CTG GAC AG |
| MMP2 Forward | GCC CCA GAC AGG TGA TCT TG |
| MMP2 Reverse | GCT TGC GAG GGA AGA AGT TGT |
| MMP9 Forward | AGA CGG GTA TCC CTT CGA CG |
| MMP9 Reverse | AAA CCG AGT TGG AAC CAC GAC |
| GAPDH Forward | GGG AAA TTC AAC GGC ACA GT |
| GAPDH Reverse | AGA TGG TGA TGG GCT TCC C |

**Customized siRNAs for targeting lncRNA:**

| **Customized siRNA name** | **Sequences (5' to 3')** |
| --- | --- |
| siRNA#1 targeting MIR4500HG003 | Sense 5'-UGU UAA GGA UCC UGG AAA AUU-3'  Antisense 5'-P UUU UCC AGG AUC CUU AAC AUU-3' |
| siRNA#2 targeting MIR4500HG003 | Sense 5'-GAU AAU AGU AAG UGA CCC AUU-3'  Antisense 5'-P UGG GUC ACU UAC UAU UAU CUU-3' |

**Antibodies used in this study**

| **Antibody** | **Source** | **Company** | **Catalog No.** | **Dilution** |
| --- | --- | --- | --- | --- |
| α-tubulin | Mouse | Sigma | T5168 | 1:5000 (WB) |
| β-actin | Mouse | Sigma | A5441 | 1:5000 (WB) |
| Ago2 | Rabbit | Abcam | ab186733 | 1:1000 (WB) |
| E-cadherin | Mouse | BD | 610182 | 1:1000 (WB) |
| Erk 1/2 | Rabbit | Cell Signaling | #9102 | 1:2000 (WB) |
| p-Erk 1/2 (Thr202/Tyr204) | Rabbit | Cell Signaling | #9101 | 1:2000 (WB) |
| GST | Mouse | Santa Cruz | sc-138 | 1:2000 (WB) |
| MMP9 | Rabbit | Abcam | ab76003 | 1:1000 (WB) |
| NF-ĸB p65 | Rabbit | Cell Signaling | #4764 | 1:2000 (WB) |
| p-NF-ĸB p65 (Ser536) | Rabbit | Cell Signaling | #3033 | 1:1000 (WB) |
