# Supplementary figures and images for: A novel long non-coding RNA MIR4500HG003 promotes tumor metastasis through miR-483-3p-MMP9 axis in triple-negative breast cancer
Source: Cell Death Dis. 2024 May 2;15(5):310. doi: 10.1038/s41419-024-06675-w (PMC11065892; doi:10.1038/s41419-024-06675-w)

Fig4C E


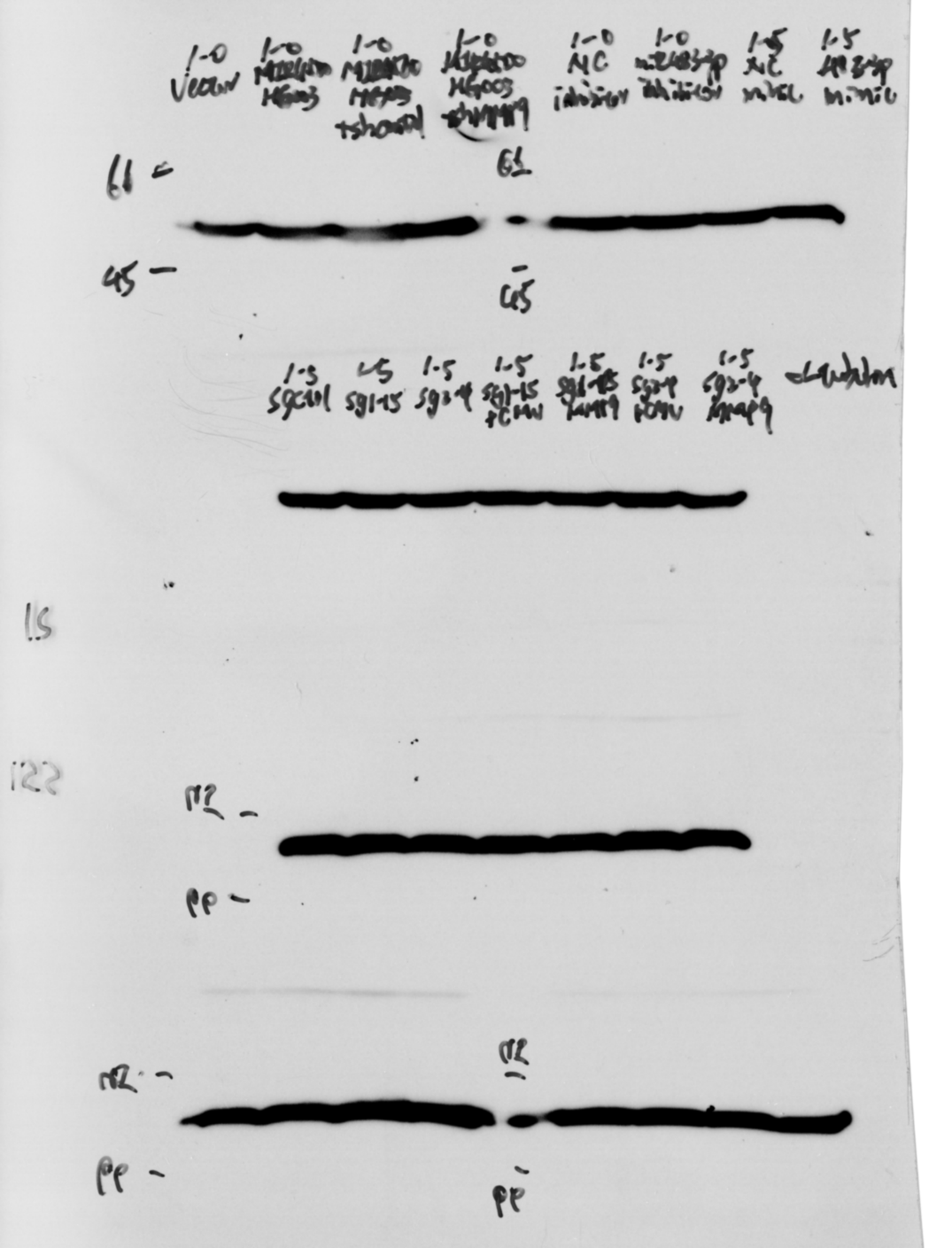

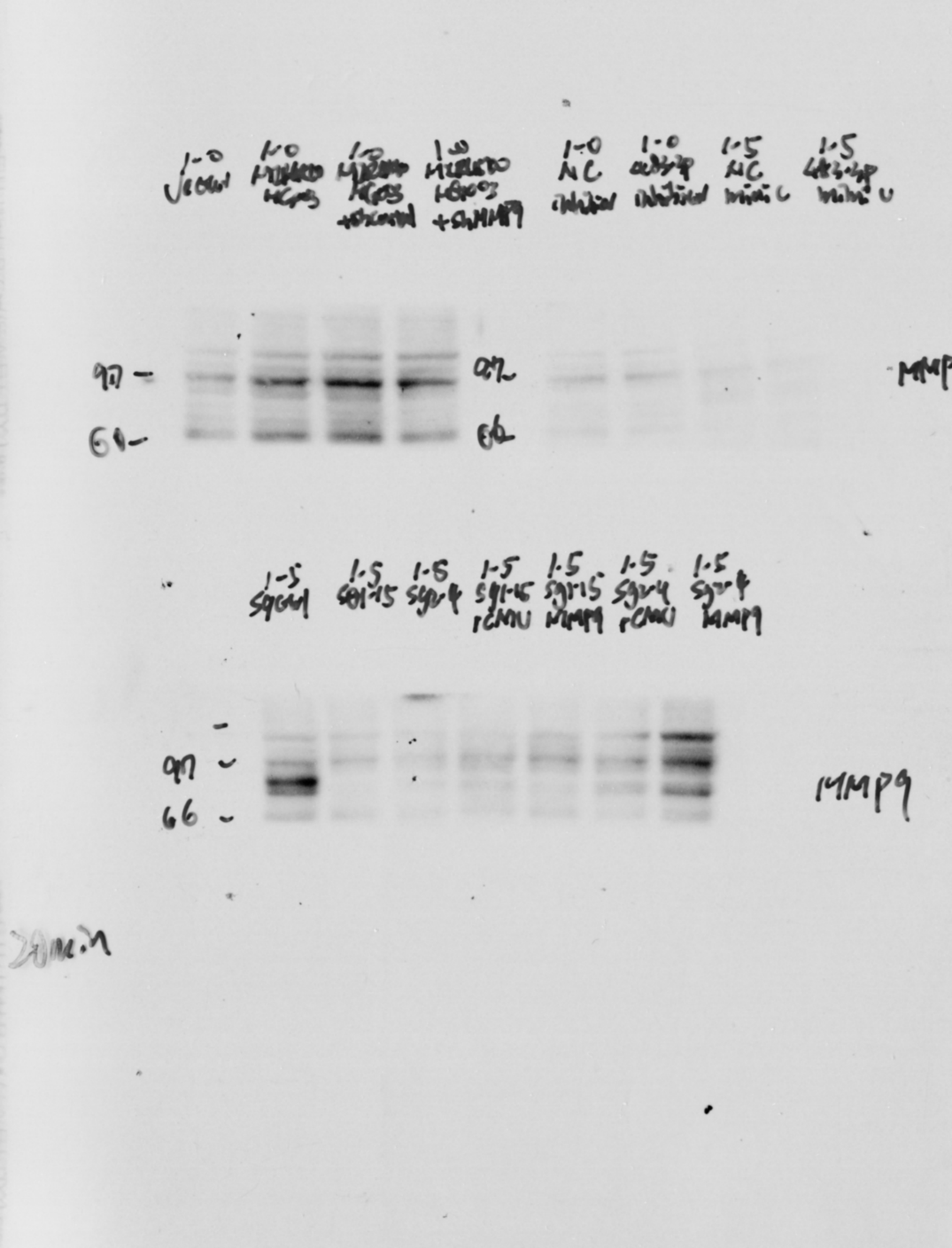


Fig5C
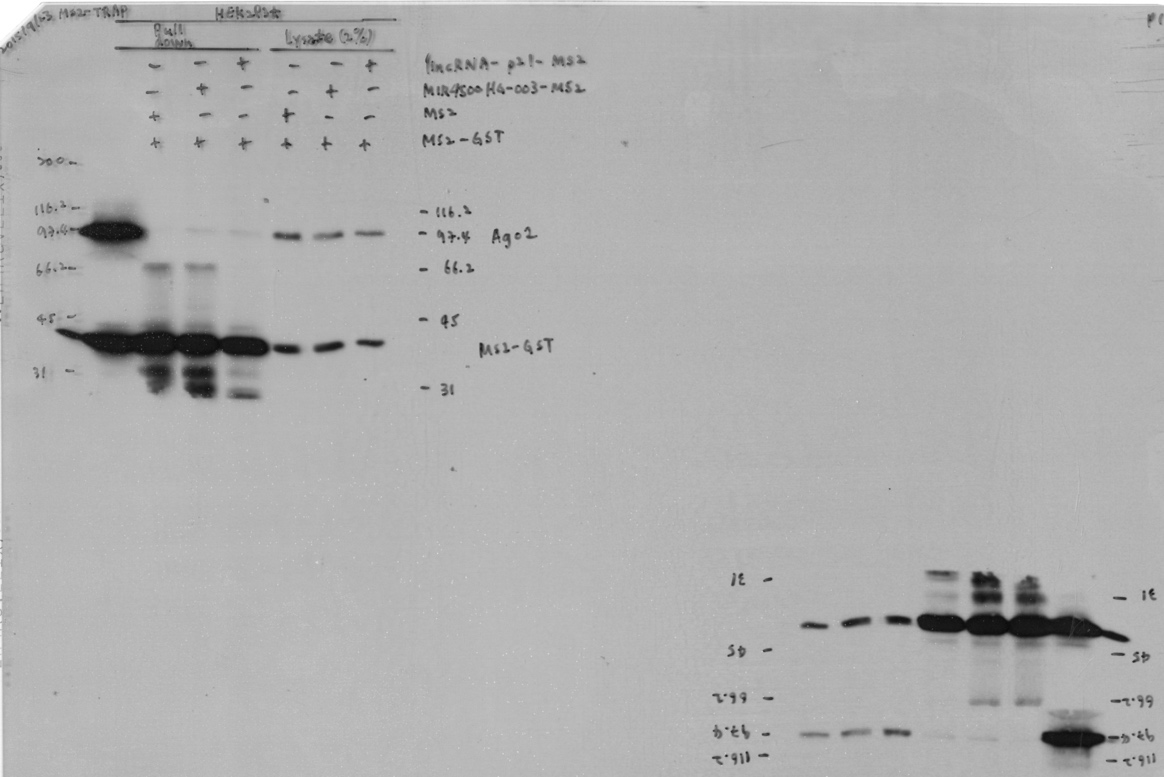

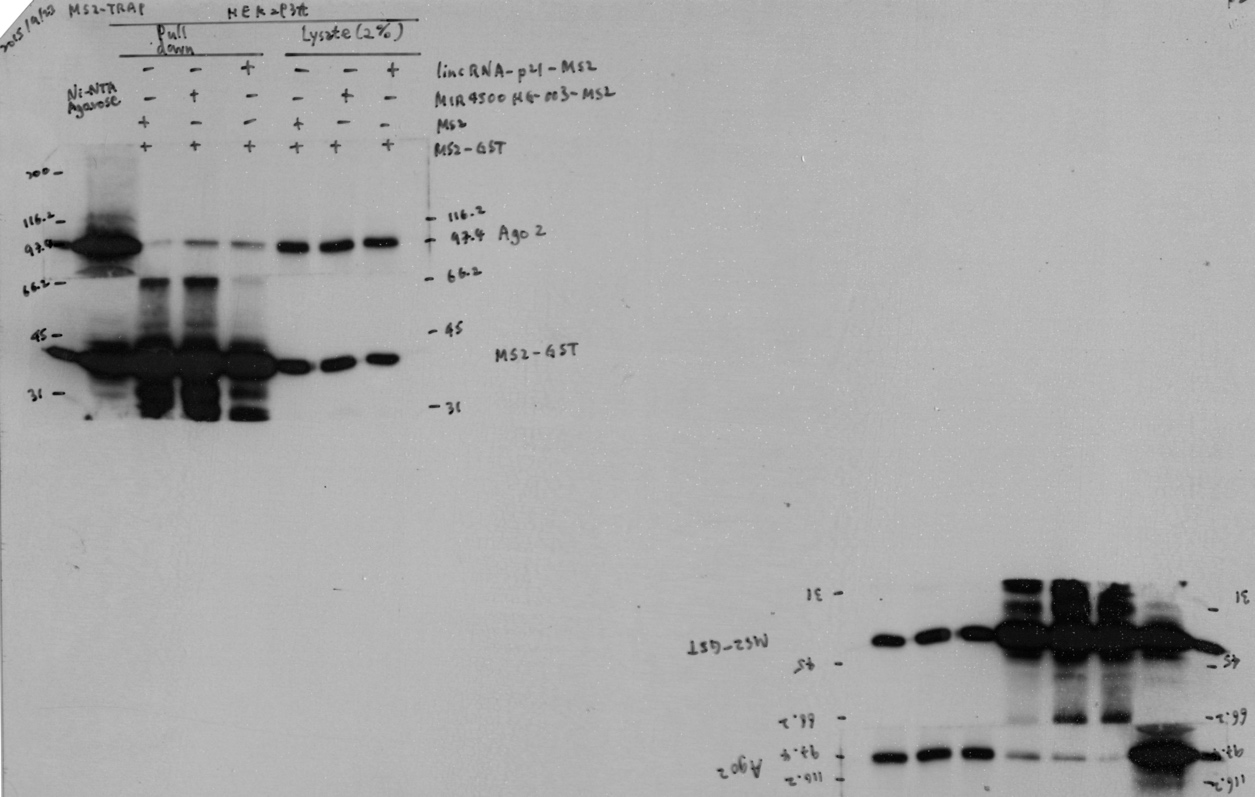


Fig 6A


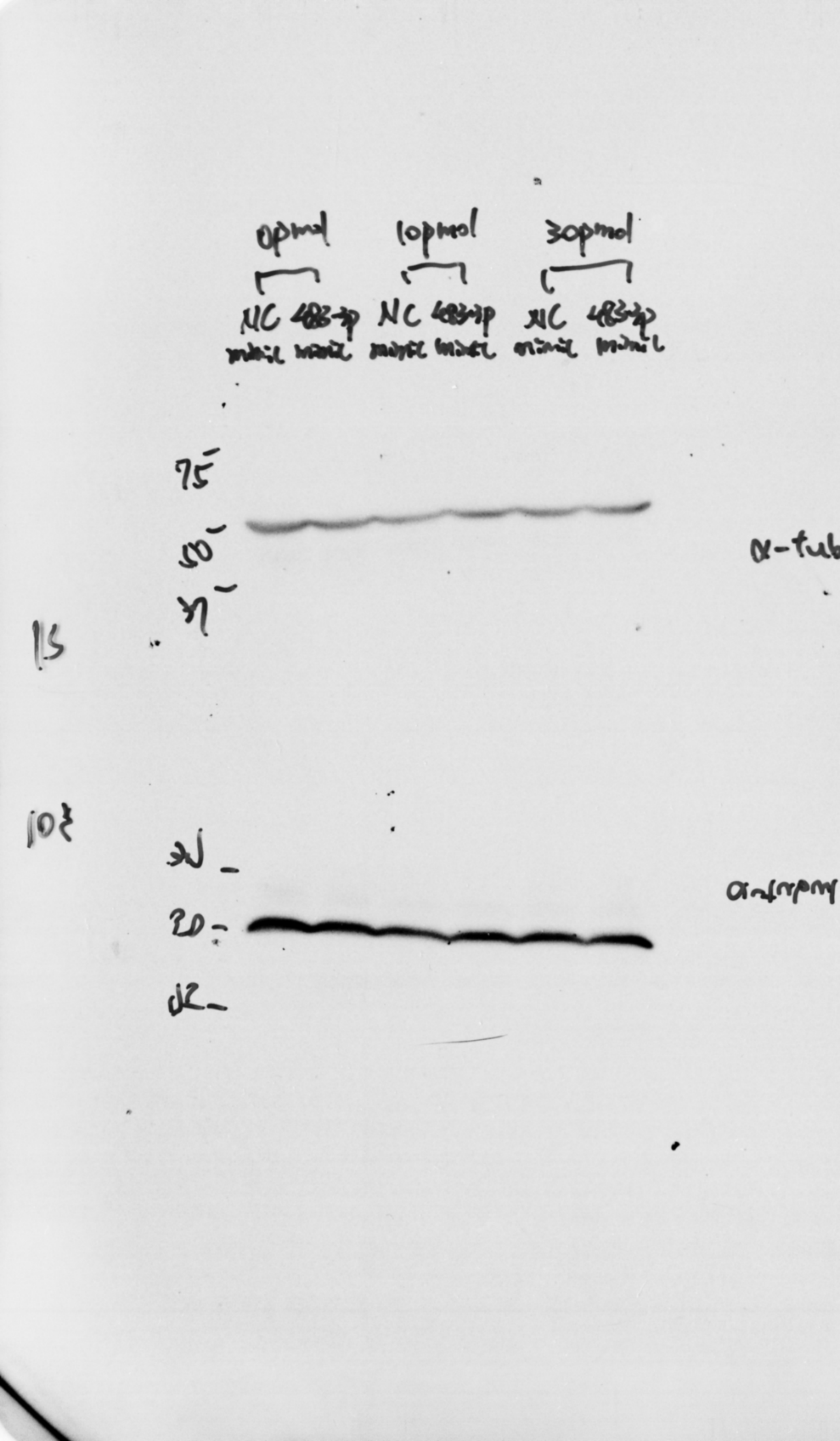

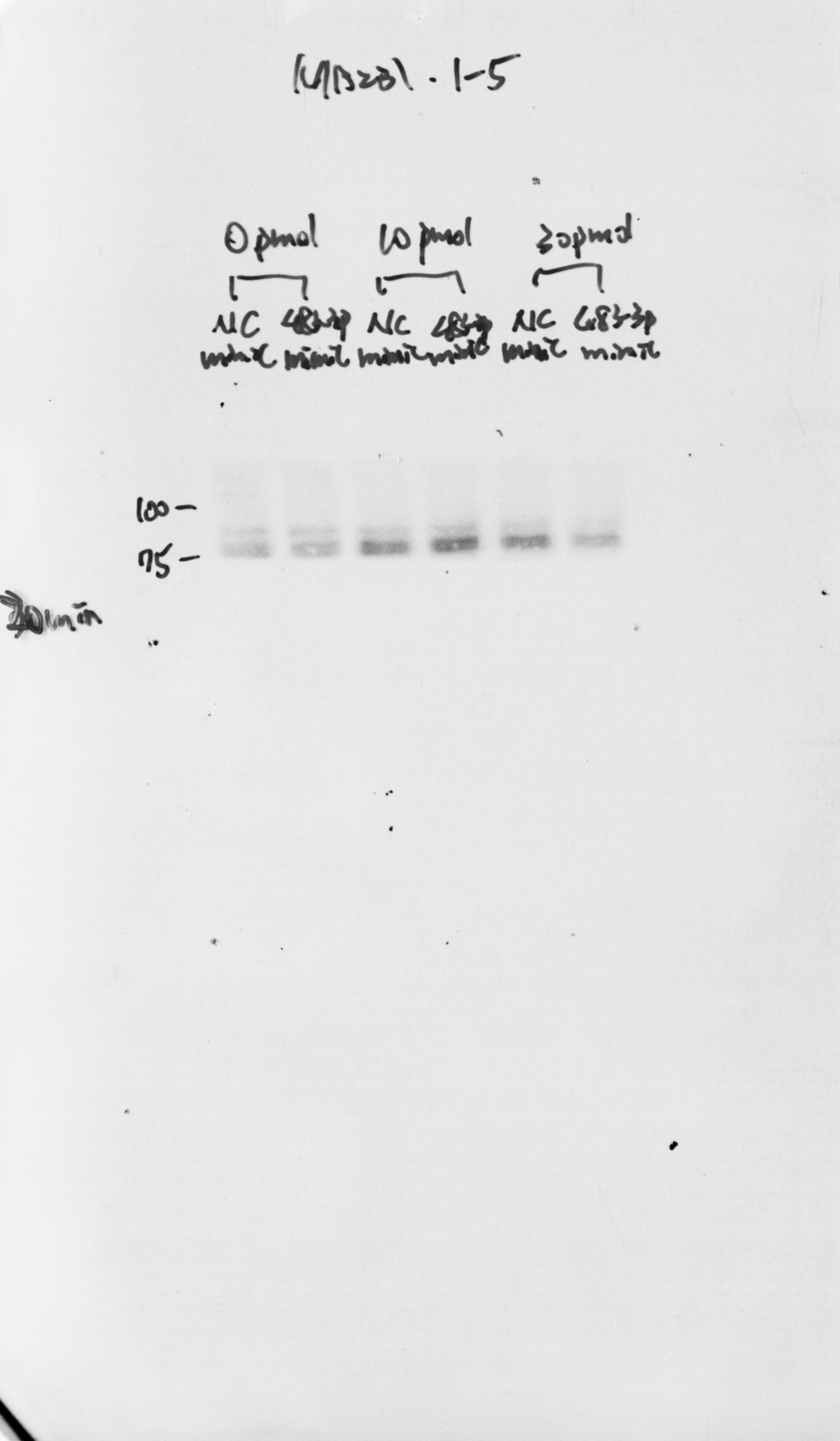


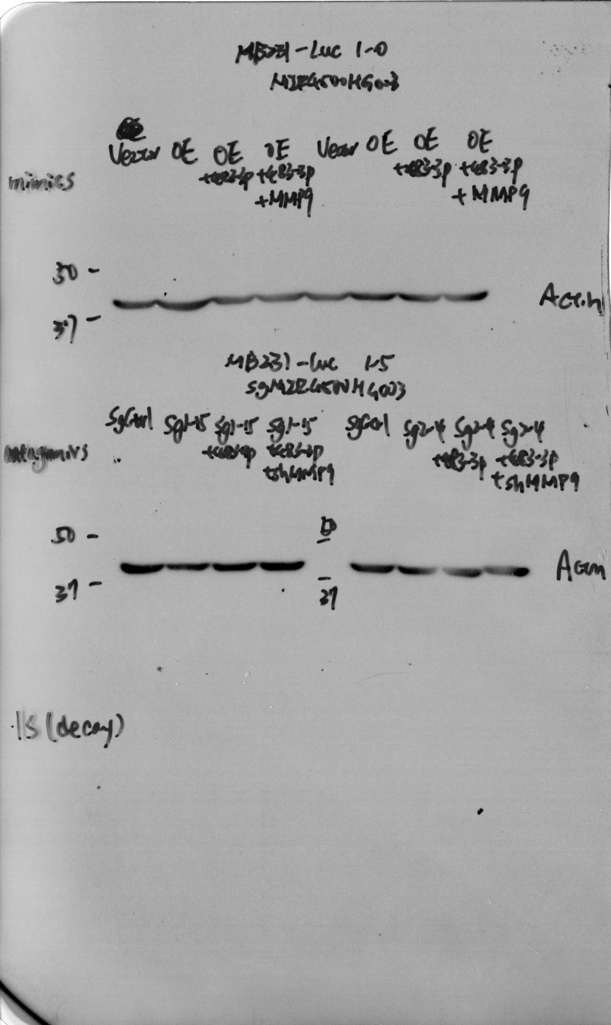

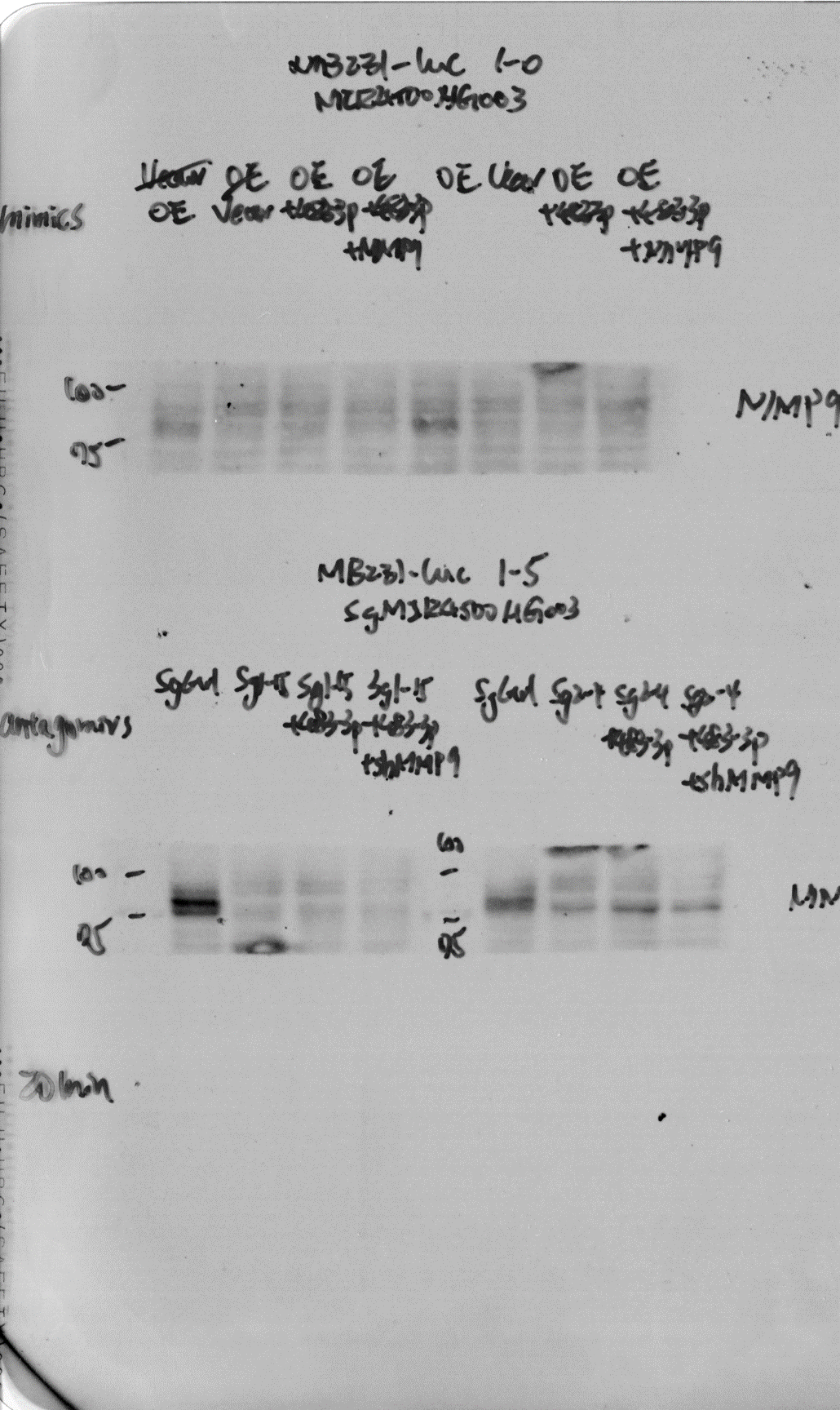

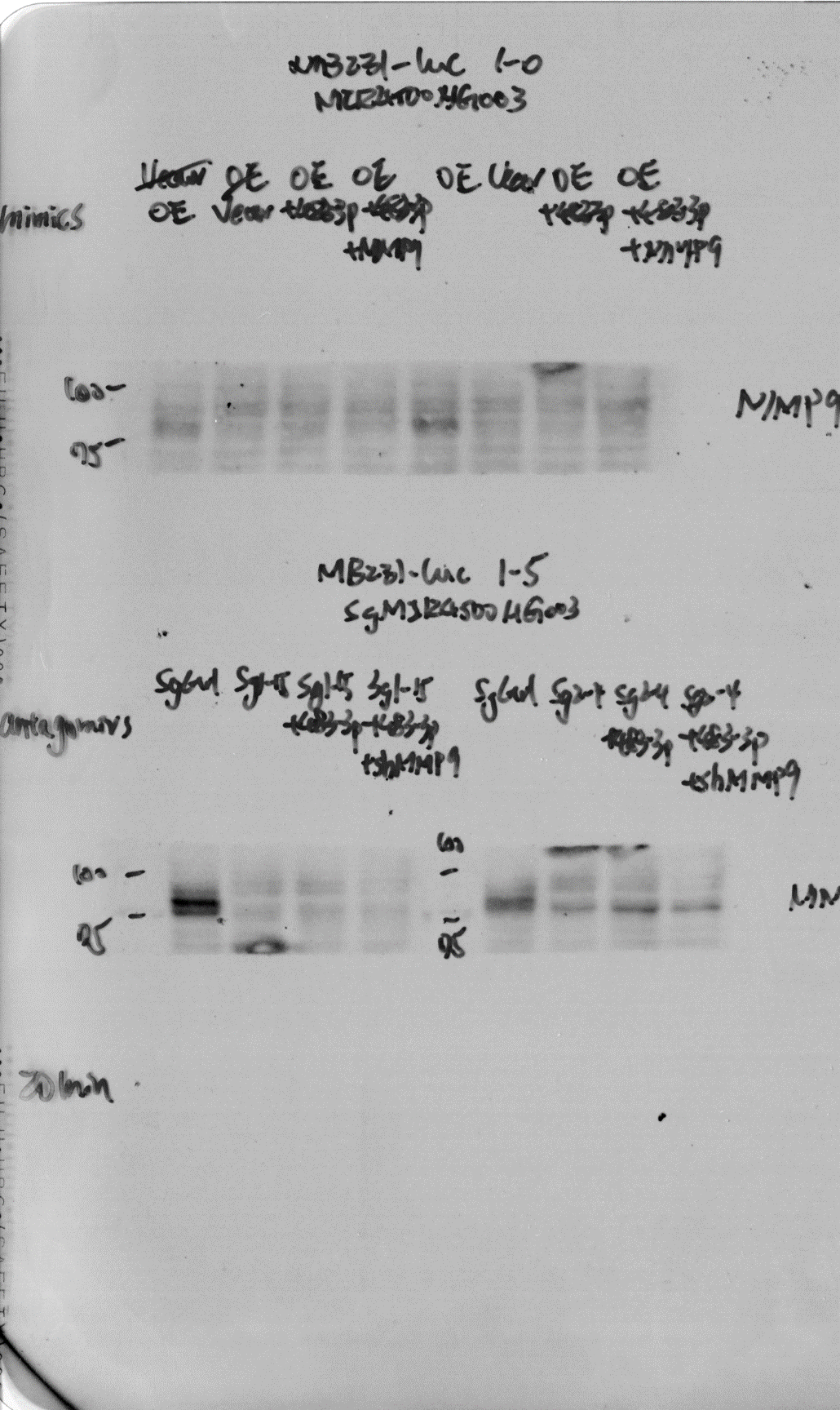
Fig 6C&E

Supplement: Supplementary file 3 — Raw data of Western blotting [file 41419_2024_6675_MOESM3_ESM.docx]
